# Printable Liquid Metal‐Textiles for Deformation‐Insensitive and Electromagnetically Robust mmWave Devices

**DOI:** 10.1002/advs.202522350

**Published:** 2026-03-19

**Authors:** Lu Ju, Buyun Yu, Rui Wang, Hao Chen, Kexin Hou, Chao Zhang, Mingyang Geng, Yunqian Dai, Yingying Zhang, Ying‐Shi Guan, Cunjiang Yu, Wei‐Bing Lu

**Affiliations:** ^1^ School of Information and Intelligent Science Donghua University Shanghai China; ^2^ State Key Laboratory of Millimeter Waves School of Information Science and Engineering Southeast University Nanjing China; ^3^ Center for Flexible RF Technology Frontiers Science Center for Mobile Information Communication and Security Southeast University Nanjing China; ^4^ School of Chemistry and Chemical Engineering Southeast University Nanjing China; ^5^ State Key Laboratory of Coordination Chemistry School of Chemistry and Chemical Engineering Collaborative Innovation Center of Advanced Microstructures Nanjing University Nanjing China; ^6^ Department of Chemistry Key Laboratory of Organic Optoelectronics and Molecular Engineering of the Ministry of Education Tsinghua University Beijing China; ^7^ Department of Electrical and Computer Engineering Department of Bioengineering Departments of Materials Science and Engineering Department of Mechanical Science and Engineering Beckman Institute for Advanced Science and Technology Materials Research Laboratory Nick Holonyak Micro and Nanotechnology Laboratory University of Illinois Urbana‐Champaign Urbana Illinois USA

**Keywords:** deformation‐insensitive, electronic textiles, liquid metal, millimeter‐wave devices, wireless communication

## Abstract

Millimeter‐wave technologies are critical to the next generation of wireless body area networks, offering high data rates an d wide bandwidths. However, realizing mechanically robust and electromagnetically stable mmWave devices remains a significant challenge due to the high sensitivity of radio‐frequency performance to conductive degradation under deformation. Here, we report a strategy to fabricate deformation‐insensitive, high‐performance mmWave electronic textiles (E‐textiles) by combining specially engineered liquid metal (LM) inks with a high‐resolution “dual‐mask” printing technique. The LM inks, composed of polyvinylpyrrolidone (PVP)‐stabilized gallium‐based nanodroplets, exhibit excellent surface compatibility, self‐healing behavior, and high conductivity (∼11.16 mΩ/sq), enabling the formation of conformal, durable circuits on textiles. We demonstrate a 26 GHz LM‐textile antenna array maintaining 9.65 dBi gain after repeated bending, as well as a microstrip transmission line with a negligible attenuation increase after mechanical cycling. Compared to printed silver inks and metallic‐cloth‐based antennas, the LM‐textile antenna exhibits superior mechanical reliability and maintains a wireless transmission range of 4.5 meters for high‐definition images. These results establish LM‐textiles as a promising platform for future wearable mmWave devices, offering scalable, flexible, and resilient solutions for high‐frequency wireless communication.

## Graphical abstract

We present a textile‐compatible, self‐healing liquid metal ink and a dual‐mask printing technique for high‐resolution patterning. Using this approach, we demonstrate high‐performance millimeter‐wave (mmWave) electronic textiles that exhibit exceptional insensitivity to deformation. This work effectively addresses the critical challenge of performance degradation in flexible mmWave devices under repeated mechanical stress, thereby paving the way for next‐generation, high‐speed wearable wireless systems.

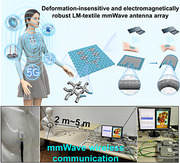

## 1 Introduction

Recent advances in Internet of Things (IoT) and wireless communication are accelerating the evolution of body area networks (BANs) into highly intelligent platforms that enable continuous, non‐contact interaction between the human body and the external environment. A wide variety of wireless wearable technologies have emerged within BANs, including human–machine interfaces, noninvasive health monitoring, and haptic communication systems [1, 2, 3, 4, 5, 6, 7, 8, 9]. To support the ongoing development of wireless BANs, millimeter‐wave (mmWave) technologies are increasingly essential due to their broader bandwidths and higher data rates, which enable scalable and robust wireless communication [10, 11, 12, 13, 14, 15, 16]. However, a critical bottleneck in wearable mmWave technologies lies in the flexibility and reliability of radio‐frequency (RF) components [17, 18, 19, 20, 21, 22, 23, 24, 25]. The degradation of electrical performance under repeated deformation is a pressing issue that must be addressed. At mmWave frequencies, RF devices are especially sensitive to metallic losses and the structural stability of conductive pathways. Even minor variations in conductivity can lead to significant electromagnetic performance degradation—such as reduced radiation gain and diminished transmission efficiency—due to the physical properties of mmWave propagation [26, 27, 28]. Consequently, it is imperative to develop mmWave devices that are both high‐performing and mechanically robust for integration into wearable systems.

E‐textiles, which offer the flexibility and breathability of traditional fabrics [29, 30, 31, 32, 33, 34], present a promising route toward seamless integration with human skin for future wearable mmWave systems. A common strategy to fabricate E‐textiles involves embedding rigid conductive materials—such as metal nanowires, conducting polymers, or carbon nanomaterials—into textile fibers [35, 36, 37, 38, 39]. However, under repeated mechanical deformation, these materials often suffer from irreversible cracking and delamination within their microscopic conductive networks [40, 41, 42], leading to substantial degradation in electrical conductivity and, consequently, in electromagnetic performance. Gallium‐based liquid metals (LMs), with their excellent electrical conductivity and intrinsic deformability, offer a compelling alternative. Their fluidic nature enables them to endure repeated mechanical stresses without significant degradation in performance [43, 44, 45, 46, 47, 48, 49, 50], making them ideal for constructing mmWave E‐textiles with robust electromagnetic properties. Yet, the high surface tension and poor interfacial compatibility of LMs with textile substrates present significant fabrication challenges [51, 52, 53, 54, 55]. Some researchers have explored forming patterned LM structures through a microchannel approach, thereby avoiding direct contact with the textiles. However, microfluidic production typically relies on multi‐step complex processing, including customized microchannel molds, pressure‐driven systems, etc. In addition, the polymer‐encapsulated LM composites are prone to electrical instability due to microchannel deformation and collapse under vertical or tensile stress [51, 52]. Therefore, to enable clothing‐compatible textile millimeter‐wave (mmWave) devices, it is still required to explore a general and effective solution for fabricating LM‐textile.

To overcome these limitations, we introduce a textile‐compatible LM ink with high conductivity and self‐healing properties, alongside a high‐resolution “dual‐mask” printing method for precision patterning. This synergistic approach enables the fabrication of mechanically durable, high‐performance mmWave devices capable of sustaining reliable electromagnetic transmission even under long‐term mechanical stress. Our LM‐textiles maintain excellent conductivity (as low as 11.16 mΩ/sq) due to the self‐healing properties of the ink, which re‐establish conductive pathways after repeated deformation. We demonstrate a 2 × 2 LM‐textile mmWave antenna array operating at 26 GHz that maintains a realized gain of 9.65 dBi after multiple bending cycles, compared to its initial gain of 10.67 dBi. Additionally, a printed microstrip transmission line shows a negligible change in attenuation after repeated deformation. Notably, the LM‐textile antenna array enables stable and precise high‐definition image transmission over a 4.5‐meter range, even after multiple bending cycles, outperforming an equivalent antenna based on commercial printed silver inks. These results underscore the exceptional mechanical durability and electromagnetic robustness of our LM‐textiles, highlighting their strong potential for next‐generation wearable wireless communication systems.

## 2 Results and Discussion

### 2.1 Design and Fabrication of LM‐Textiles

**FIGURE 1:**
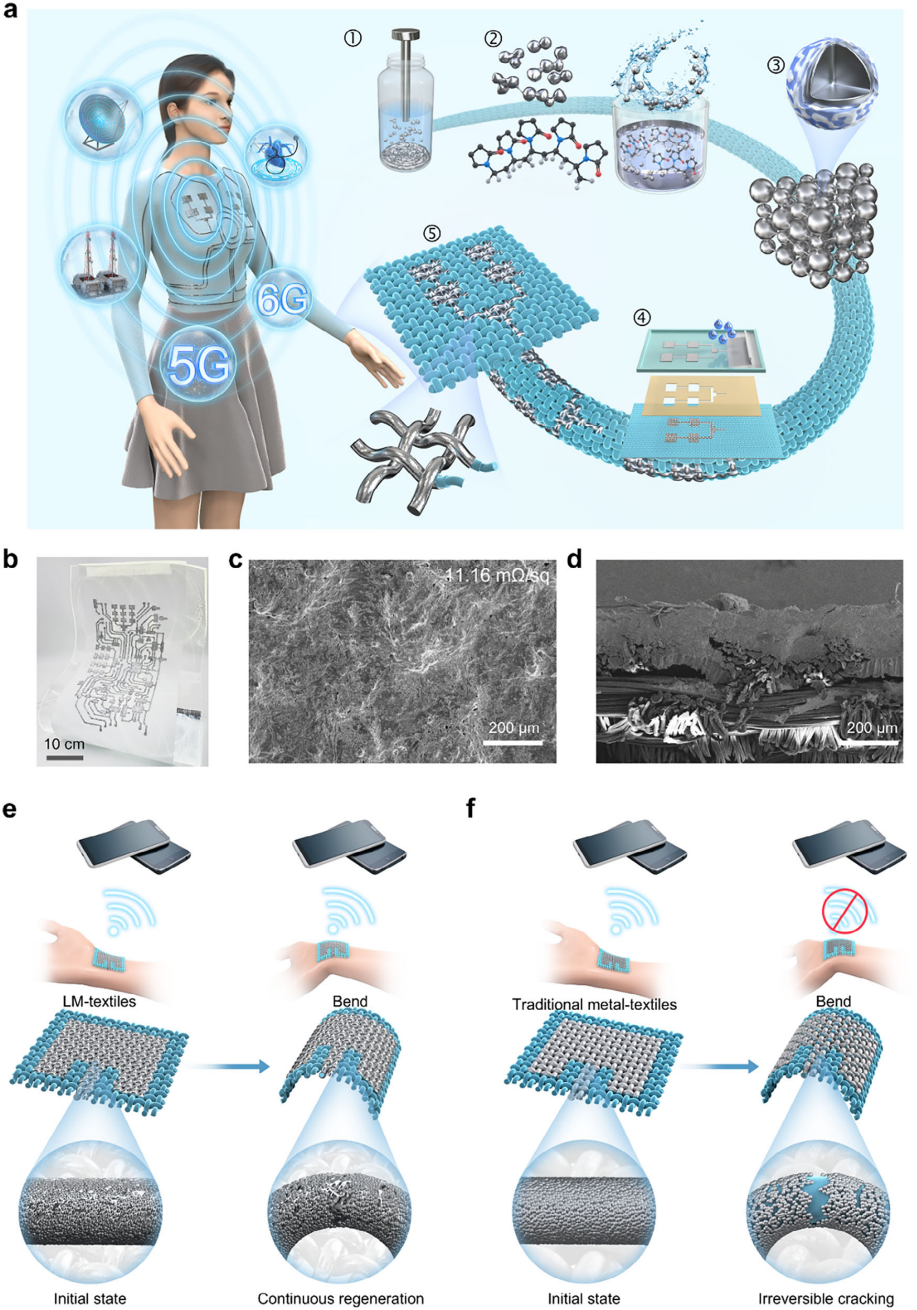
Deformation‐insensitive and electromagnetically robust mmWave devices enabled by LM‐textiles. (a) The design mechanism and preparation processes of the LM‐textile mmWave devices, and the future applications of the system. (b) Demonstration of the flexible LM‐textile circuit. (c,d) SEM images of the (c) surface morphology, and (d) cross‐section of the printed LM‐textile circuit after mechanical tests. (e,f) Schematic comparison of (e) flexible functionalized liquid metal (LM) nanodroplets with (f) traditional conductive nanodroplets, demonstrating how conventional materials suffer wireless disconnection under cyclic mechanical stress due to irreversible structural damage.

The high conductivity, self‐healing characteristic, and printability of LMs offer high potential for developing high performance and mechanically robust mmWave E‐textiles in future wireless communication (Figure 1a). However, the LMs exhibit poor adhesion, leading to limitations in constructing functional devices and systems with high‐precision patterns. Here, to overcome this challenge, we specially designed LM inks to fabricate LM‐textile mmWave devices. The whole design principle and manufacturing process are schematically illustrated in Figure 1a. Briefly, to improve their processability and printability, we take a top‐down method to synthesize LM inks. First, after a prolonged probe sonication, the pristine LM droplets are fragmented into nanodroplets that demonstrate a variety of nanoscale and surface effects, due to the formed gallium oxide (Ga_2_O_3_) layer (Figure S1a,b). However, LM nanodroplets without surface modification (LM nanodroplets) still show poor interfacial compatibility with textiles, resulting in imprecise and discontinuous circuits. To further enhance the wettability and printability, PVP with biocompatibility characteristics serves as a stabilizer and dispersant of the LM nanodroplets (Figure S2). The PVP‐stabilized LM nanodroplets (functionalized LM nanodroplets) formed via surface modification exhibit excellent affinity with textiles, thereby enabling compatibility with printing technologies. We respectively take stencil printing and screen printing to prepare LM‐textile circuits. The morphology and cross‐sectional of the two printed patterns are investigated via scanning electron microscopy (SEM). SEM analysis indicates that the electrical conductivity of the stencil‐printed circuits is superior to that of screen‐printed circuits, owing to enhanced infiltration of functionalized LM nanodroplets into the textile matrix (Figures S3–S5). Thus, we propose a “dual mask” technique that combines the advantages of stencil and screen printing, which can attain well‐define patterns with 150 (±58.2) µm resolution (Figures S6 and S7). In general, a laser‐engraved heat‐release tape stencil (serving as the first mask) is laminated onto the textile substrate to define screen printing region. Then, the functionalized LM nanodroplets are uniformly and precisely printed on the confined area by screen printing (serving as the second mask), thereby forming the LM‐textiles with high patterning accuracy and excellent electrical conductivity. As shown in Figure 1b, a high‐precision and complex LM‐textile pattern was created by the synergistic integration of specially functionalized LM nanodroplets and printing technologies. The flexible LM‐textile can arbitrarily conform to various irregular curved surfaces and remains high‐performance and stable conductivity. SEM images present that functionalized LM nanodroplets remain densely adhered to the textile surface and maintain a uniform average sheet resistance of 11.16 mΩ/sq after repeated 5000 repeated bending cycles (Figure 1c,d; Figure S8). Compared with conventional conductive materials, the highly conductive and robust electrical characteristics enable functionalized LM nanodroplets to address the challenges of low metallic loss and electromagnetic stability in RF devices (Figure 1e; Table S1). Due to non‐fluid and rigid nature, traditional conductive materials are prone to fracturing at bending state (Figure 1f). The irreversible cracks in conductive pathways will greatly degrade the conductivity of E‐textiles and may even cause complete failure. In mmWave devices, even a slight decrease in conductivity will inevitably lead to pronounced distortions in the electromagnetic performance. In contrast, flexible functionalized LM nanodroplets can continuously re‐establish electrical connections and sustain high conductivity during continuous rupture and regeneration processes of Ga_2_O_3_ layers caused by external force (Figure 1e) [53]. Therefore, the LM‐textile with intrinsic flow [53] and self‐healable features offers a potential solution for achieving high‐performance, electromagnetic‐robust mmWave devices.

### 2.2 Interaction between LM Nanodroplets and Textiles

**FIGURE 2:**
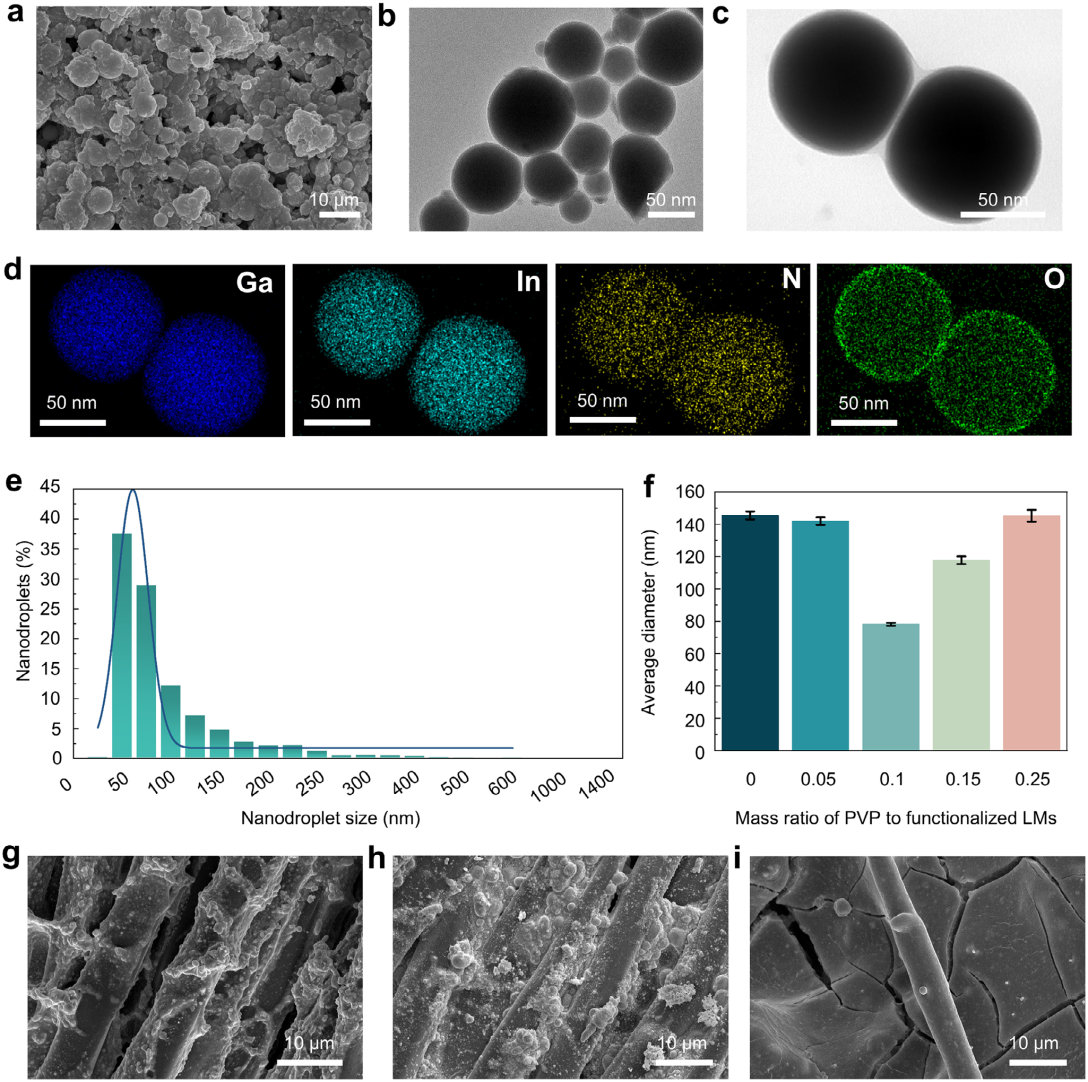
Characterization of LM inks. (a) SEM, (b,c) HRTEM, and (d) the corresponding EDS analysis images of the functionalized LM nanodroplets (functionalized LMs). (e) Size distribution and (f) average size of the functionalized LMs under different PVP concentrations (Mean ± SEM). The weight ratios of PVP to functionalized LMs are 0, 0.05, 0.1, 0.15, and 0.25, respectively. (g–i) SEM images of functionalized LMs printed on textile substrates under (g) optimal, (h) low, and (i) high concentrations of PVP.

The morphology and size distribution of nanodroplets are crucial for controlling the stability, fluidity, and printability properties of LM inks. The LM nanodroplets without surface modification show structural instability, readily lead to re‐aggregation. As a long‐chain structure, PVP can create steric hindrance between LM nanodroplets, thereby improving the colloidal stability and suppressing aggregation. Herein, LM inks are developed, consisting of functionalized LM nanodroplets stabilized and dispersed by PVP. SEM image illustrates that functionalized LM nanodroplets are formed by encapsulating spherical LM nanodroplets—acting as the core—with uniform PVP nanolayers (Figure 2a–c). The stable interactions between the PVP and LM nanodroplets are attributed to the bonding process illustrated in Figure S9. The polar groups in the molecular structure of PVP (carbonyl and nitrogen atoms) form coordination bonds with Ga (III) on the surface of gallium oxide layer, thereby creating a stable PVP nanolayer on the surface of LM nanodroplets. In Figure 2b,c, high resolution transmission electron microscopy images (HRTEM) reveal that uniformly dispersed functionalized LM nanodroplets mostly exhibit regular spherical shapes and smooth surfaces, and an ultra‐thin Ga_2_O_3_ layer of approximately 3 nm was formed (Figure S10). The uniformly wrapped functionalized LM nanodroplets exhibit enhanced elasticity and the adjacent nanodroplets are closely connected by the PVP nanolayers (Figure 2c). These results strongly confirm that the functionalized PVP nanolayer effectively modifies the fluidity and acts as a physical barrier between adjacent LM nanodroplets, thereby preventing their aggregation. Moreover, EDS analysis is investigated in Figure 2d. The presence of nitrogen (N) and oxygen (O) further confirms the attachment of PVP nanolayers on the surface of the LM nanodroplets. Additionally, the distribution of O element indicates the existence of a Ga_2_O_3_ nanolayer. The Ga and O elements are homogeneously distributed within functionalized LM nanodroplets, which illustrates the nanodroplets maintain excellent stability without phase separation following high‐intensity ultrasound. The Fourier transform infrared (FTIR) spectroscopy apparently identify absorption peaks corresponding to C─H, C═O, and C─N stretching vibrations, demonstrating the interaction between PVP nanolayers and LM nanodroplets (Figure S11). In Figure S12, X‐ray photoelectron spectroscopy (XPS) analysis reveals the emergence of N 1 s peak at 399.84 eV, and the Ga 3 d peaks show discernible shifts as result of the surface modification of the PVP on the Ga_2_O_3_. These results demonstrate that the introduction of PVP significantly alters the local chemical environment of N and Ga, leading to a bonding interaction between PVP and the LMs.

As observed in the microscopic morphological characterization above, PVP nanolayer plays a vital role in preparing the functionalized LM nanodroplets with superior dispersibility and colloidal stability. In Figure 2e,f, the functionalized LM nanodroplets exhibit narrow size distribution with an average diameter of approximately 78.18 nm. It is notable that the liquidity of the functionalized LM nanodroplets can be reduced by maintaining a small, uniform, and regular spherical morphology, to form high‐resolution circuit patterns [53]. As shown in Figures 1c and 2g, the fabricated functionalized LM nanodroplets exhibit excellent surface compatibility and printability with textile, allowing densely stacked nanodroplets to firmly adhere to the textile substrate and build a seamless 3D conductive network. In Figure S13a,b, SEM images show the unmodified LM nanodroplets transform into powdery particles that merely float on the textile surface. It should be noted that a large number of unmodified LM nanodroplets fail to establish good contact with textile substrate and aggregate on textile surface, resulting in an almost non‐conductive LM‐textile. PVP concentration is optimized to ensure electrical conductivity and printing resolution. At a low concentration of PVP, insufficient surface modification leads to agglomeration of adjacent LM nanodroplets and non‐uniform attachment on textile substrate (Figure 2h; Figure S14). It is denoted that some LM nanodroplets still exhibit a powdery morphology, hindering the formation of an effective conductive pathway. The broader size distribution also suggests that LM nanodroplets cannot be uniformly packed, resulting in agglomeration at low concentrations (Figure S15). With increasing PVP concentrations, the average size of the functionalized LM nanodroplets decreases (Figure 2f). Excessive PVP concentration causes a broadened size distribution and some larger agglomeration (>1000 nm) (Figure 2f; Figure S16a,b). These results may be attributed to the fact that excess PVP layers not only bind to the surface of LM nanodroplets but also undergo self‐polymerization and agglomeration. As revealed by SEM images, the surface morphology of LM‐textile became smoother. Some PVP agglomeration and large cracks appeared in the conductive pathways, disrupting electrical continuity. (Figure 2i; Figure S17).

**FIGURE 3:**
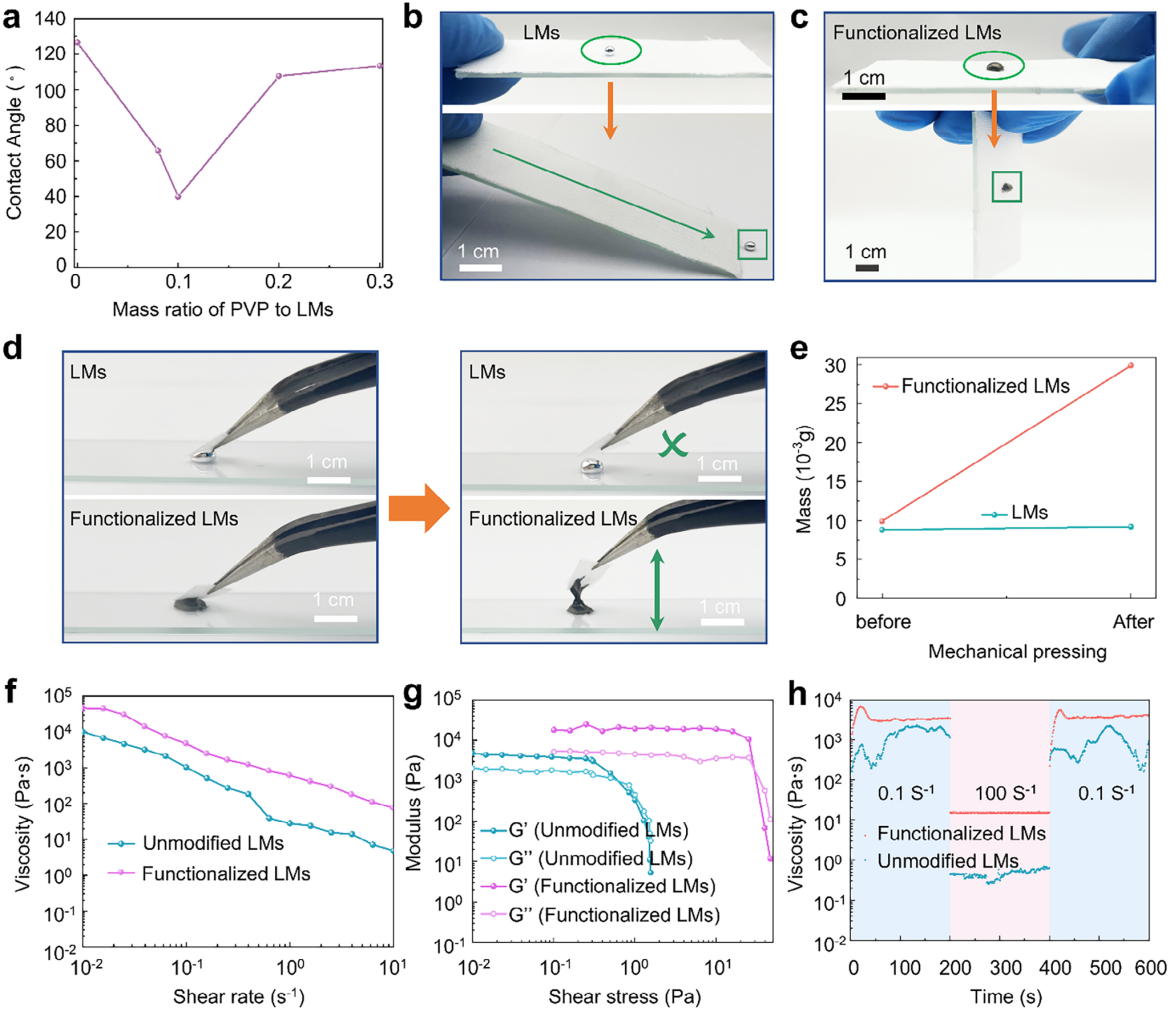
The adhesion and rheological properties characterization. (a) Contact angles of large pristine LM droplets (LMs) and functionalized LM nanodroplets (functionalized LMs) at different PVP concentrations. The weight ratios of PVP to functionalized LMs are 0, 0.08, 0.1, 0.2, and 0.3, respectively. (b,c) Photographs of (b) large pristine LMs and (c) functionalized LMs on textile substrate supported by glass plates. (d,e) Comparison of (d) adhesion properties and (e) mass retention between pristine liquid metal (LM) and functionalized LM on textile substrates. (f–h) Rheological characterization of unmodified and functionalized liquid metal (LM) nanodroplets: (f) viscosity versus shear rate, (g) storage (G′) and loss (G″) moduli as functions of shear stress, and (h) thixotropic behavior.

Large pristine LM droplets exhibit a high surface tension, resulting in poor affinity with textile substrate. As shown in Figure 3a and Figure S18a, the large pristine LM droplet attached on the textile exhibits a contact angle (CA) of ∼126.7°. Through ultrasonication, the formed Ga_2_O_3_ nanolayers improve the wettability between LM nanodroplets and textile substrate (Figure S18b). However, the formed LM nanodroplets show irregular and unstable morphology, which hinders their extrusion into complex and high‐precision patterns. The modification of PVP nanolayers further improves the surface compatibility between the functionalized LM nanodroplets and textile substrate (Figure S18c–f). The contact angle between functionalized LM nanodroplets and textile substrate decrease to 39.8° under optimal synergistic interaction (Figure 3a; Figure S18d). The contact angle is obviously smaller compared to that of large pristine LM droplets on the textile substrate. To further verify the interfacial adhesion, a large pristine LM droplet and functionalized LM nanodroplets were respectively positioned on textile substrate supported by glass plates (Figure 3b,c). Significantly, the large pristine LM droplet instantly rolled off the textile substrate when the inclination angle reached 30° (Figure 3b). Conversely, the functionalized LM nanodroplets still keeps stationary on the textile substrate even at an inclination angle of 90° (Figure 3c). In Figure 3d, the large pristine LM droplet and functionalized LM nanodroplets are directly deposited onto glass plate. It is evident that almost no residue from the pristine LM droplet remains on the textile, whereas the functionalized LM nanodroplets exhibit strong adhesion to the textile. In addition, the mass of functionalized LM nanodroplets attached to textile is approximately 50 times greater than that of the large pristine LM droplet (Figure 3e). The ultrasonication and PVP modification endow the functionalized LM nanodroplets with the surface and nanoscale effects, including enhanced interfacial adhesion and reduced intrinsic fluidity, which improve their suitability for high‐precision printing.

Appropriate rheological properties are of great significance for high‐precision printing technologies. However, compared to large pristine LM droplets, the small size and weak interactions of LM nanodroplets after ultrasonication generally lead to poor rheological properties. The synergistic interaction nanoscale between dimensions and PVP modification improves the rheology of functionalized LM nanodroplets. In Figure 3f, the functionalized LM nanodroplets exhibit typical shear thinning characteristics, which are crucial for the continuous extrusion in screen printing technology. The functionalized LM nanodroplets show a significantly superior tunable shear viscosity. A large G′/G″ ratio highlights the elasticity‐dominant role in the rheology properties of the printed inks. In Figure 3g, the functionalized LM nanodroplets produce a larger G′/G″ ratio and higher yield stress, allowing the printed nanodroplets to retain their shape and avoid spreading over the textile during printing process. Figure 3h describes the thixotropic performance of the LM nanodroplets. The results demonstrate that the functionalized LM nanodroplets show excellent response, recovery, and stability, which are significantly superior LM nanodroplets without PVP modification. The excellent viscosity and elasticity of the functionalized LM nanodroplets can support a rapid and precise prototyping of complex patterns, satisfying the printing requirements during screen printing process. The rheological properties of the functionalized LM nanodroplets are sensitive to the PVP concentration, as shown in Figure S19a. The lager G″ indicates elasticity performance of the functionalized LM inks is broken (Figure S19b).

Through nano‐materialization and surface modification treatments, functionalized LM nanodroplets achieve enhanced surface affinity with textile. The strong contact between functionalized LM nanodroplets and textiles produces robust LM‐textiles with a average sheet resistance of 11.16 mΩ/sq, which is sufficient to support high‐performance electromagnetic transmission of mmWave devices. For mmWave devices, both structural accuracy and electrical conductivity are indispensable for ensuring electromagnetic performance. In this paper, the specially designed LM inks possess typical shear thinning behavior and tunable viscosity. These features endow functionalized LM nanodroplets with excellent printability, demonstrating significant potential in high‐precision mmWave devices.

### 2.3 High‐Performance and Electromagnetic‐Robust LM‐Textile mmWave Devices

**FIGURE 4:**
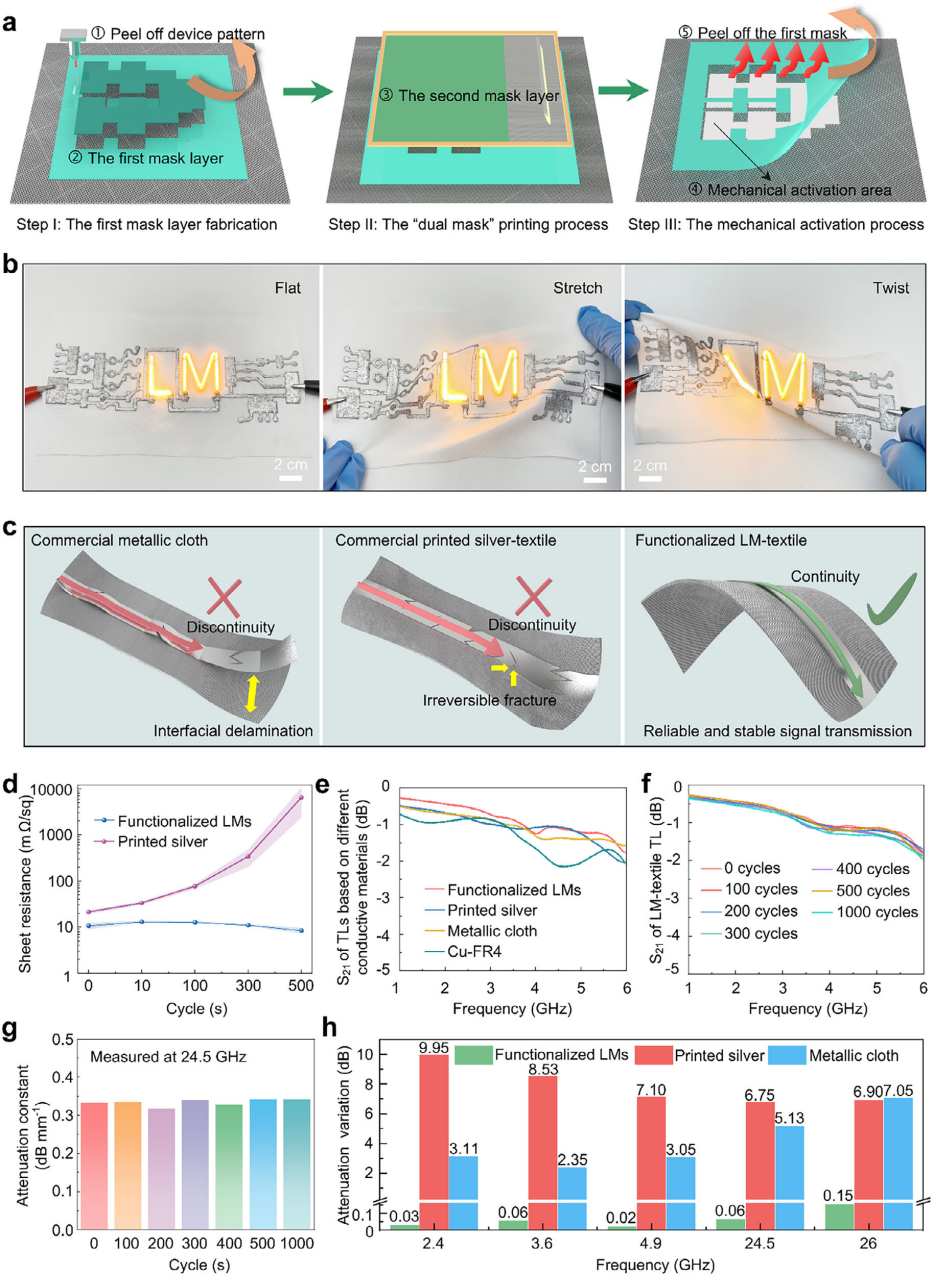
The fabrication, electrical properties, and electromagnetic properties characterization of the LM‐textile devices. (a) The manufacturing process of the LM‐textile mmWave devices. (b) Photographs of an LM‐textile LED array under flat, stretched, and twisted states. (c) Electromagnetic wave transmission of microwave TLs based on different E‐textiles when subjected to external forces. (d) Average sheet resistance of the LM‐textile and printed silver‐textile as a function of repeated bending cycles. Data presented as mean ± SE, *n* = 3 (e) Measured insertion loss of microstrip TL based on functionalized LM nanodroplets (functionalized LMs) and other conductive materials in Sub‐6G. (f) Measured insertion loss of LM‐textile microstrip TL in Sub‐6G after repeated mechanical tests. (g) At 24.5 GHz, measured attenuation constant of LM‐textile microstrip TL after repeated mechanical tests. (h) The attenuation variation of microstrip TLs based on LM‐textile and other conductive textiles after 1000 cycles bending.

Material loss, patterning precision, and mechanical reliability are crucial for realizing high‐performance mmWave E‐textiles. Owing to the intensified skin effect in conductors at mmWave frequency band, metallic loss can greatly affect the electromagnetic performance of RF devices. By substituting solid conductive materials with metal‐like and robust functionalized LM nanodroplets, the fabricated LM‐textile mmWave devices overcome the challenges related to metallic loss and mechanical reliability. The efficiency of mmWave devices is also susceptible to the dielectric loss of the substrate. We conduct electromagnetic properties measurements on various textiles with different weave pattern and density, followed by choosing high‐density woven polyester textile with a low dielectric loss (ε_r_ = 1.6 and δ = 0.009, Figure S20) as the dielectric substrate. Compared to RF devices operating in lower frequencies, mmWave devices pose much stricter requirements for structure accuracy due to shorter wavelengths. Slight structural mismatch will degrade the transmission performance and radiation efficiency of mmWave devices. To address these challenges, we propose the “dual mask” printing method to precisely fabricate high‐performance mmWave devices, including microstrip transmission lines and mmWave antenna arrays. The three‐step manufacturing procedure of the LM‐textile mmWave devices is demonstrated in Figure 4a. First, to construct the first mask layer for stencil printing, we utilize a high‐precision laser cutting machine to accurately cut the device structure from the heat release tape laminated onto the textile substrate. In general, the high‐throughput stencil printing technique allows sufficient functionalized LM nanodroplets deposition onto the textile surface, but high permeability inevitably leads to nanodroplet diffusion. Screen printing technology can achieve high‐precision patterns, but overly dense masks limit permeability. Thus, to achieve high‐performance LM‐textile, it is essential to make a trade‐off between nanodroplets throughput and patterning resolution. As the first mask layer, the laser‐engraved stencil adhered to the textile defines the area for screen printing. Screen printing stencil with relatively high density serve as the second mask layer to control the permeability during the printing process. This strategy integrates the advantages of both printing techniques by moderately reducing mask density while effectively suppressing nanodroplet diffusion at the structure edges during printing process. Moreover, during the mechanical activation process, the first mask layer restrains the fractured Ga_2_O_3_ layer from drifting freely, thereby protecting the printed patterns. Finally, the heat‐release tape stencil automatically peels off from the substrate at 100°C (Figure S21) and removes the excess nanodroplets and fractured Ga_2_O_3_ layer, followed by forming LM‐textile mmWave devices. As shown in Figure 4b, through the “dual mask” printing method, we fabricated LM‐textile LED light‐emitting circuits. The printed LED array maintains a clear and bright luster under bending, stretching, and twisting. In addition, the reliability of the electrical connection is crucial for the long‐term stability of the microwave devices. We used the an “interposer” strategy to connect the LM‐textile circuits to external circuits (Figure S22), which is compatible with conventional high‐temperature soldering processes.

The combination of laser cutting and transfer techniques has been widely adopted as a mature approach for fabricating high‐performance textile microwave devices [29, 30, 33, 34]. However, the resulting composite structure typically relies on the lamination of separate functional textile layers bonded by adhesives, rather than forming a fully integrated conductive‐textile system. In Figure 4c, the composite‐textile microwave devices are highly susceptible to suffer an interfacial delamination under large deformation, which severely disrupts impedance matching. Especially for mmWave devices, poor impedance matching will greatly decrease transmission efficiency. The integrated structure formed by printing onto textiles can effectively prevent interfacial delamination. Nevertheless, traditional printed metal materials cannot withstand bending and twisting, owing to the inherently rigid characteristics. After repetitive strain, irreversible cracks inevitably occur in conductive pathways of textile microwave devices based on traditional printed metal materials (Figure 4c). The significantly increased metallic loss will cause a severe deterioration in the transmission efficiency of mmWave devices. Thus, high‐performance and deformation‐insensitive conductive E‐textiles are crucial for realizing efficient and stable mmWave devices. In our strategy, the intrinsic low‐modulus and liquidity enables the fabricated highly conductive LM‐textile to withstand various deformations. In Figure 4d and Video S1, supporting information, the average sheet resistance of the LM‐textile changes from an initial 10.67 to 8.4 mΩ/sq, indicating well‐maintained conductivity after being repeated bending. For comparison, we perform bending experiments on commercial printed silver‐textile. The silver‐textile exhibited a significant decrease in electrical performance, due to the severely fractured conductive pathway (Video S2). To further evaluate the mechanical robustness of the LM‐textiles under long‐term wear, we conducted repeated mechanical tests, including cyclic bending with a bending radius of 7.5 mm at a rate of 5 mm/s. In Figure S23a,b, the LM‐textile exhibits a negligible change in electrical conductivity (1.375 Ω/cm) even after 10,000 cyclic bending tests, which remains nearly consistent with the initial value of 0.8 Ω/cm. In addition, we performed a twisting experiment on the LM‐textiles. As shown in S24a,b (supporting information), over 10,000 twisting cycles, the LM‐textiles maintain stable electrical resistance of 1.525 Ω/cm. In contrast, the silver‐textile is severely broken. Therefore, metal‐like and ultra‐robust LM‐textiles can support a reliable and stable electromagnetic transmission of mmWave devices under various mechanical deformations.

Microstrip transmission lines (TL) are fundamental components in microwave devices and systems. The transmission characteristics of microstrip lines are highly sensitive to metallic losses, making them an ideal platform for evaluating the electromagnetic performance of metallic materials. We fabricated a 30 mm‐length LM‐textile microstrip TL (Figure S25) and conducted detailed experiments on its electromagnetic performance and durability (Figure 4e–h; Figures S26–S29). At 2.45 GHz, this 30 mm‐length LM‐textile microstrip TL exhibited a low average insertion loss of only 0.73496 dB, demonstrating excellent electromagnetic performance. The insertion losses of other microstrip TLs are 0.82, 0.78, and 0.85 dB, respectively. It is evident that the LM‐textile microstrip TL exhibits electromagnetic performance comparable to those based on traditional metal materials, which can be attributed to its excellent electrical conductivity and low metallic loss characteristics.

Slight metallic loss and structural damage can drastically reduce the electromagnetic efficiency of mmWave devices due to their physical properties, such as the shorter wavelength, and high propagation loss. In general, functionalized LM nanodroplets can realize effective regaining of conductive pathways by rupturing the oxide layer within structural network of LM‐textiles. The self‐healable nature makes LM‐textiles to recover their conductive networks during repeated deformations (Figure S27). Thus, the deformation‐insensitive characteristic endows LM‐textile microstrip TL line with reliable and robust electromagnetic transmission efficiency under repeated mechanical stress. At 24.5 GHz, the average attenuation constant of LM‐textile microstrip TLs is 0.35806 dB mm^−1^ after 1000 bending cycles, which is consistent with original average value of 0.35092 dB mm^−1^ (Figure 4g; Figure S29). In contrast, as exhibited in Figure 4h, the printed silver inks and metallic‐cloth‐based microstrip TLs exhibit a sharply increased attenuation of 6.75 dB and 5.13 dB at 24.5 GHz, while their transmission efficiency decreases by more than 50% at 26 GHz. It can be observed that the external force severely degrades the transmission performance of conventional metal‐based microstrip TLs, whereas the deformation‐insensitive nature of LM‐textile devices ensures robust electromagnetic performance.

**FIGURE 5:**
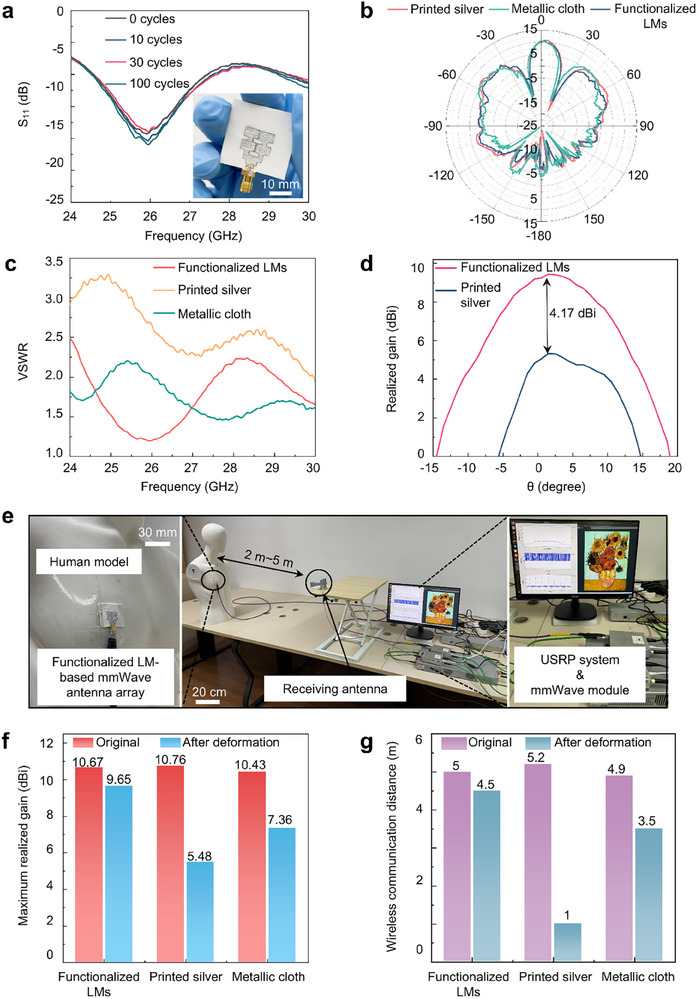
Electromagnetic‐mechanical properties characterization of the LM‐textile mmWave antenna arrays. (a) Measured S_11_ of the 2 × 2 LM‐textile mmWave antenna array under original state and after repeated bending cycles. (b) Measured radiation pattern of mmWave antenna arrays based on LM‐textile, printed silver‐textile, and metallic cloth. (c) Voltage Standing Wave Ratio (VSWR) mmWave antenna arrays based on LM‐textile, printed silver‐textile, and metallic cloth when subjected multiple mechanical bending cycles. (d) Measured radiation pattern of mmWave antenna arrays based on LM‐textile and printed silver‐textile when subjected multiple mechanical bending cycles. (e) Wireless communication experiments based on a USRP system. Electromagnetic performance comparison of 2 × 2 mmWave antenna arrays. (f) Maximum realized gains and (g) wireless communication distances for LM‐textile and printed silver‐textile arrays in original states and after mechanical strain.

Building on the specially designed LM‐textiles, we created a 2 × 2 mmWave antenna array for 5G wireless communication and future BAN (Figure S30). To assess the electromagnetic performance, we measured its S‐parameters and radiation pattern (Figure S31). The LM‐textile mmWave antenna array exhibits good impedance matching from 25 to 27 GHz (Figure 5a). The measured maximum realized gain of the antenna reaches 10.67 dBi (Figure 5b), indicating high radiation efficiency. Compared to printed silver inks and metallic‐cloth‐based mmWave antenna (Figure S32a,b), the proposed LM‐textile mmWave antenna array exhibits comparable electromagnetic properties (Figure 5b; Figure S33). Notably, the LM‐textile maintains superior electrical performance even after mechanical deformation. During durability testing, the antenna array was subjected to repeated bending cycles. As shown in Figure 5a,c, the LM‐textile mmWave antenna array maintains stable impedance matching and minimal power loss within acceptable variations, enabling the high‐efficiency signal and power transmission required for wearable mmWave applications. In contrast, external forces disrupt the microscopic conductive pathways in printed silver‐textile antennas, leading to high Voltage Standing Wave Ratio (VSWR) and significant signal reflection (Figure 5c). Figure S34 demonstrates the LM‐textile mmWave antenna conforming to a cylindrical foam substrate (15 cm diameter). Radiation pattern measurements confirm consistent performance after repeated deformation (Figure 5d; Figure S35), whereas the printed silver‐textile antenna suffers severe radiation efficiency degradation under similar conditions, revealing its poor mechanical durability. The metallic‐cloth‐based antenna array exhibits substantial electromagnetic distortion, including frequency shifts and wireless disconnection, caused by conductive layer delamination from the textile substrate after bending cycles (Figure S36). In summary, we employ a top‐down method approach combined with surface modification to fabricate functionalized LM nanodroplets, which show enhanced compatibility and adhesion with textile substrates even under repeated deformations. However, LM‐textiles still face the risk of leakage when subjected to long‐time external mechanical stimuli, such as friction, large deformations. Therefore, it is still a challenge to identify effective encapsulation techniques for LM‐based next‐generation wearable mmWave devices. To improve structural and electromagnetic stability under prolonged exposure to friction, temperature variations, moisture, and biofluids, we implemented polydimethylsiloxane (PDMS) encapsulation [18]. This chemically stable, waterproof protective layer enables the LM‐textile devices to maintain stable and reliable electromagnetic performance after multiple rigorous washing and friction tests (Figures S37 and S38). A comprehensive comparison of mmWave devices using different conductive materials and fabrication methods is provided in Table S2. Our LM‐textile mmWave devices combine superior electromagnetic performance with exceptional robustness, showing great promise for future body area networks (BANs).

To verify the reliable and robust radiation efficiency of the fabricated LM‐textile mmWave antenna array, we carried out wireless communication experiments. The experimental setup consisted of a universal software radio peripheral (USRP) system serving as a mmWave wireless communication base station, a standard horn antenna serving as the receiving antenna, a human body model, and our prepared LM‐textile mmWave antenna array. The LM‐textile mmWave transmitting antenna was mounted on the chest of the human model and connected to the USRP system's transmitting port via a coaxial line. The standard horn receiving antenna was connected to the USRP's receiving port. In addition, we set the modulation scheme of the USRP system to binary phase shift keying (BPSK). As demonstrated in Figure 5e and Video S3, the LM‐textile mmWave antenna array enabled precise and stable wireless transmission of high‐definition images over a 5‐meter communication range. We further tested the antennas under mechanical strain. The LM‐textile mmWave antenna array maintained a maximum realized gain of 9.65 dBi (Figure 5f), ensuring stable signal transmission over 4.5 meters due to its self‐healing capability (Figure 5g). In contrast, the printed silver‐textile antenna array exhibited a significantly reduced maximum realized gain of 5.48 dBi, leading to substantially degraded wireless transmission range. Moreover, to further substantiation regarding real‐word applicability of LM‐textile antennas to mmWave BANs, we simulated S_11_, realized gain, the SAR value of the LM‐textile antenna array conformed to the human body phantom in CST 2022 software. The electromagnetic model and simulated results are exhibited in Figures S39 and S40. These results show that the LM‐textile antenna array exhibits a slight frequency shift at bending state, yet maintains good impedance matching and achieve a average realized gain of 8.8 dBi at 26 GHz (Figure S39; Table S3). Even under these conditions, the simulated SAR values are still below the standard limit of 2.0 W/kg (Table S3). These results demonstrate that the LM‐textile mmWave antenna array possesses excellent mechanical robustness and reliability, maintaining stable electromagnetic performance even when subjected to various mechanical deformations during prolonged daily use.

## 3 Conclusion

In summary, we demonstrate a highly conductive, mechanically resilient, and electrically self‐healing LM‐textile network for the construction of high‐performance, deformation‐insensitive mmWave devices. Leveraging the intrinsic metallic conductivity and regenerative properties of gallium‐based LM nanodroplets, our LM‐textiles address the stringent requirements of mmWave systems, including low metallic loss, high structural precision, and robust electromagnetic performance, beyond what is typically achievable in lower‐frequency RF devices. We engineered functionalized LM inks with excellent surface compatibility and tunable rheological properties, enabling scalable fabrication on textile substrates. Through an innovative dual‐mask printing strategy that combines the throughput of stencil printing with the precision of screen printing, we achieved high‐resolution patterns with strong substrate adhesion and minimal diffusion. This process supports precise impedance matching and structural fidelity essential for mmWave operation. Crucially, the self‐healing behavior of the LM network allows for repeated mechanical deformation without compromising device performances. Our wireless communication experiments demonstrate that the LM‐textile mmWave antenna arrays maintain stable and high‐quality signal transmission even under strain, conforming to curved surfaces such as the human body. Collectively, the LM‐textile platform introduced here establishes a promising pathway for constructing next‐generation wearable mmWave devices, offering mechanical durability, electromagnetic robustness, and scalable manufacturability essential for the future of high‐frequency body area networks and soft electronics.

## 4 Experimental Section

### 4.1 Materials

Liquid metal (74.5% Ga and 24.5% In by weight) was purchased from Dongguan Hua Titanium Material Technology Co., Ltd, China. PVP powers, ethanol and isopropanol were purchased from Aladdin Reagent Co., Ltd, China. The textile substrate was obtained from Xuanjin Clothing Co., Ltd, China. The textiles for printing LM inks were supplied by Yucheng Fabric Co., Ltd, China. The printed silver inks (JY 50) were purchased from Shanghai Julong Electronic Technology Co. Ltd.

### 4.2 Preparation of LM Inks

First, 1 g pristine LM bulk droplet was added to 1 mL of ethanol. The mixture was ultrasonicated for 1 h using an ultrasonic homogenizer (XM‐900 T, Xiaomei ultrasonic instrument, Co., Ltd, Kunshan) to achieve the LM nanodroplets. The ethanol was completely removed through evaporation and drying at 100°C, yielding LM powder that was collected from the substrate. For ink preparation, the LM powder was uniformly dispersed in isopropanol with PVP through additional ultrasonication (XM‐400UVF, Xiaomei ultrasonic instrument, Co., Ltd, Kunshan).

### 4.3 Preparation of Microstrip Transmission Lines and 2 × 2 mmWave Antenna Arrays

The microstrip transmission line based on Cu‐FR4 was fabricated by traditional PCB technology (Shenzhen JLC Technology Group Co., Ltd). The 2 × 2 mmWave antenna array based on commercial printed silver inks was prepared by screen printing technology. The 2 × 2 mmWave antenna array based on metallic cloth was fabricated by laser cutting (Dazu Yueming Laser Cutting Co., Ltd.) and transfer technologies.

### 4.4 Morphology and Electrical Characterization

The surface and cross‐section morphologies of the LM‐textiles were characterized by SEM (FEI Nova Nano SEM450, USA). The FEI Tecnai G20 was used to observe microstructure of LM nanodroplets. High resolution transmission electron microscopy (HRTEM) and energy dispersive X‐ray spectroscopy (EDS) mapping was performed by Talos F200X. The sheet resistance of the LM‐textiles was measured by a four‐point probe (HPS2526, Changzhou Helpass Electronic Technology Co., Ltd). The resistance was measured using a digital multimeter (Fluke).

### 4.5 Dielectric Characteristic Measurement of the Textile Substrate

A split post dielectric resonator (QWED F‐SPDR‐5.1) was used to measure the dielectric characteristics of the textile substrate. The resonator analyzes the dielectric characteristics of the inserted textile substrate by evaluating the shift in resonant frequencies and Q factor compared to an empty resonator. The resonant frequency and Q factor of the split post dielectric resonator were achieved by a vector network analyzer (Transcom T5260C).

### 4.6 Electromagnetic Simulations

Full‐wave simulation performed in CST 2022 microwave studio was conducted for designing the structures and researching the electromagnetic performance of microwave devices, such as impedance matching performance, radiation performance and electromagnetic field distributions. Ansys HFSS was used to designed and simulated the electromagnetic performance of the power divider and mmWave antenna arrays.

### 4.7 Rheological Properties Characterization

The rheological behaviors of LM inks were evaluated by TA Instruments DHR‐2 system equipped with a 25 mm‐diameter flat plate and a 2.5 mm gap. Viscosity measurements were performed at a shear rate of 0.01 to 10 S^−1^. To achieve the G′ and G″ of the LM inks and PVP solution, the oscillatory measurements were conducted at a frequency of 1 Hz, while the shear stress was set to 0.01 to 100 Pa. In addition, the cross‐point of the G′ and G″ curves represent the yield stress of the LM inks. To evaluate the thixotropy properties of the LM inks, the viscosity measurements were carried out at a shear rate of 0.1, 100, and 0.1 S^−1^, respectively.

### 4.8 Electromagnetic Measurements of Microstrip Transmission Lines and mmWave Antenna Arrays

A vector network analyzer (Keysight N5227B) was used to measure the S‐parameters of microstrip transmission lines and mmWave antenna arrays. The realized gain of the mmWave antenna arrays was tested in an anechoic chamber in Southeast University. In the bend‐state measurements, the mmWave antenna arrays were conformed to cylindrical foams with a diameter of 15 cm to evaluate their RF performance stability.

### 4.9 Wireless Sensing and Communication Measurements of the mmWave Antenna Arrays

Wireless communication experiments were performed by a USRP system (Luowave N310‐LW, Wuhan), a standard horn antenna (DZN‐SGHA‐220‐20‐K, Nanjing Guanjun Technology. CO., LTD), a human body model, and our proposed mmWave antenna arrays. The USRP system was conducted for simulating a mmWave wireless communication base station. The fabricated mmWave antenna arrays serve as a transmitting antenna and were connected to the transmitting port of the USRP system through a coaxial line. The standard horn antenna with a realized gain of 21 dBi served as a receiving antenna was connected to the receiving port of the USRP system. The center frequency and output power of the USRP system were set as 26 GHz and 0 dBm. The EIRP is 21 dBm. The receiver sensitivity of receiver is ‐100 dBm.

### 4.10 Mechanical Performance Tests

Mechanical testing was performed using a tensile tester (Flextest, Hunan Nanoup Electronics Technology Co., Ltd.) to investigate the resistance variation of textile electrodes under cyclic strain. To further validated the mechanical durability, manual bending and stretching operation were conducted on LM‐textiles. In the mechanical durability testing, cyclic bending and stretching were performed on the limited area of the E‐textiles, followed by measuring the sheet resistance of the limited area using a four‐ point probe.

## Conflicts of Interest

The authors declare no conflicts of interest.

## Supporting information




**Supporting File 1**: advs74739‐sup‐0001‐SuppMat.docx.

**Supporting File 2**: advs74739‐sup‐0002‐VideoS1.mp4.

**Supporting File 3**: advs74739‐sup‐0003‐VideoS2.mp4.

**Supporting File 4**: advs74739‐sup‐0004‐VideoS3.mp4.

## Data Availability

The data that support the findings of this study are available from the corresponding author upon reasonable request.

## References

[1] H. U. Chung , B. H. Kim , J. Y. Lee , et al., “Binodal, Wireless Epidermal Electronic Systems With in‐Sensor Analytics for Neonatal Intensive Care,” Science 363 (2019): eaau0780, 10.1126/science.aau0780. 30819934 PMC6510306

[2] X. Yu , Z. Xie , Y. Yu , et al., “Skin‐Integrated Wireless Haptic Interfaces for Virtual and Augmented Reality,” Nature 575 (2019): 473–479, 10.1038/s41586-019-1687-0. 31748722

[3] S. Niu , N. Matsuhisa , L. Beker , et al., “A Wireless Body Area Sensor Network Based on Stretchable Passive Tags,” Nature Electronics 2 (2019): 361–368, 10.1038/s41928-019-0286-2.

[4] M. Mahmood , D. Mzurikwao , Y.‐S. Kim , et al., “Fully Portable and Wireless Universal Brain–Machine Interfaces Enabled by Flexible Scalp Electronics and Deep Learning Algorithm,” Nature Machine Intelligence 1 (2019): 412–422, 10.1038/s42256-019-0091-7.

[5] H. Sheng , L. Jiang , Q. Wang , et al., “A Soft Implantable Energy Supply System That Integrates Wireless Charging and Biodegradable Zn‐Ion Hybrid Supercapacitors,” Science Advances 9 (2023): adh8083. 10.1126/sciadv.adh8083 PMC10651135 37967195

[6] J. Zhan , W. Lu , C. Ding , et al., “Flexible and Wearable Battery‐Free Backscatter Wireless Communication System for Colour Imaging,” npj Flexible Electronics 8 (2024): 1–12.

[7] S. Patel , Z. Rao , M. Yang , and C. Yu , “Wearable Haptic Feedback Interfaces for Augmenting Human Touch,” Advanced Functional Materials 36 (2025): 2417906.

[8] J. Kim , J. Park , Y.‐G. Park , et al., “A Soft and Transparent Contact Lens for the Wireless Quantitative Monitoring of Intraocular Pressure,” Nature Biomedical Engineering 5 (2021): 772–782, 10.1038/s41551-021-00719-8. 33941897

[9] J. Song , H. Ryu , W. Bai , et al., “Bioresorbable, Wireless, and Battery‐Free System for Electrotherapy and Impedance Sensing at Wound Sites,” Science Advances 9 (2023): ade4687. 10.1126/sciadv.ade4687 PMC9946359 36812305

[10] H. Tataria , M. Shafi , A. F. Molisch , M. Dohler , H. Sjöland , and F. Tufvesson , “6G Wireless Systems: Vision, Requirements, Challenges, Insights, and Opportunities,” Proceedings of the IEEE 109 (2021): 1166–1199, 10.1109/JPROC.2021.3061701.

[11] J. Kimionis , A. Georgiadis , S. Daskalakis , and M. Tentzeris , “A Printed Millimetre‐Wave Modulator and Antenna Array for Backscatter Communications at Gigabit Data Rates,” Nature Electronics 4 (2021): 439–446, 10.1038/s41928-021-00588-8.

[12] W. Hong , Z. Jiang , C. Yu , D. Hou , H. Wang , and C. Guo , “The Role of Millimeter‐Wave Technologies in 5G/6G Wireless Communications,” IEEE Journal of Microwaves 1 (2021): 101–122, 10.1109/JMW.2020.3035541.

[13] X. He , B. Tehrani , R. Bahr , W. Su , and M. Tentzeris , “Additively Manufactured Mm‐Wave Multichip Modules With Fully Printed “Smart” Encapsulation Structures,” IEEE Transactions on Microwave Theory and Techniques 68 (2020): 2716–2724, 10.1109/TMTT.2019.2956934.

[14] T. Rappaport , Y. Xing , G. MacCartney , A. Molisch , E. Mellios , and J. Zhang , “Overview of Millimeter Wave Communications for Fifth‐Generation (5G) Wireless Networks—With a Focus on Propagation Models,” IEEE Transactions on Antennas and Propagation 65 (2017): 6213–6230, 10.1109/TAP.2017.2734243.

[15] A. Pellegrini , A. Brizzi , L. Zhang , et al., “Antennas and Propagation for Body‐Centric Wireless Communications at Millimeter‐Wave Frequencies: A Review [Wireless Corner],” IEEE Antennas and Propagation Magazine 55 (2013): 262–287, 10.1109/MAP.2013.6645205.

[16] G. Sacco and M. Zhadobov , “Physical Interactions between Millimeter Waves and Human Body: From Macro‐ to Micro‐Scale,” IEEE Journal of Microwaves 4 (2024): 318–328, 10.1109/JMW.2024.3407712.

[17] M. Wagih , G. Hilton , A. Weddell , and S. Beeby , “Broadband Millimeter‐Wave Textile‐based Flexible Rectenna for Wearable Energy Harvesting,” IEEE Transactions on Microwave Theory and Techniques 68 (2020): 4960–4972, 10.1109/TMTT.2020.3018735.

[18] L. Ju , B. Yu , H. Chen , et al., “Multilevel Printed Wearable Radio‐Frequency Intelligent Identification Platform for Object Recognition,” ACS Applied Materials & Interfaces 16 (2024): 49856–49867, 10.1021/acsami.4c06404. 39230937

[19] L. Ju , Z. Liu , B. Yu , H. Chen , Z. Xiao , and W. Lu , “Stretchable and Dynamically Tunable Attenuator Based on Graphene,” IEEE Transactions on Microwave Theory and Techniques 70 (2022): 2999–3008, 10.1109/TMTT.2022.3164708.

[20] F. Wang , J. Zhou , Y. Li , and X. Xu , “Millimeter‐Wave Patch Antennas With Ultralow Profile of 20 µm on Flexible Substrate,” IEEE Transactions on Antennas and Propagation 71 (2023): 2339–2349, 10.1109/TAP.2023.3241359.

[21] H.‐Q. Wang , B.‐Y. Yu , Z.‐Y. Huang , et al., “Shape‐Adaptive and Recyclable Radio‐Frequency Devices Based on Polymer with Variable Stiffness,” Cell Reports Physical Science 5 (2024): 101882, 10.1016/j.xcrp.2024.101882.

[22] Z.‐H. Chen , H. Chen , B.‐Y. Yu , et al., “Flexible and Stretchable Microwave Filter Based on Substrate Integrated Plasmonic Waveguide,” IEEE Transactions on Microwave Theory and Techniques 72 (2024): 5970–5982, 10.1109/TMTT.2024.3381156.

[23] L. Sun , R. Wang , B. Yu , et al., “High‐gain Millimeter‐Wave Stretchable Array Antenna Based on Electrospun‐Batio_3_/PDMS Composite Membrane Substrate,” ACS Applied Materials & Interfaces 17 (2025): 35938–35949. 40468167 10.1021/acsami.5c06206

[24] T. Chang , Y. Tanabe , C. C. Wojcik , et al., “A General Strategy for Stretchable Microwave Antenna Systems Using Serpentine Mesh Layouts,” Advanced Functional Materials 27 (2017): 1703059, 10.1002/adfm.201703059.

[25] J. Zhu , J. Fox , N. Yi , and H. Cheng , “Structural Design for Stretchable Microstrip Antennas,” ACS Applied Materials & Interfaces 11 (2019): 8867–8877, 10.1021/acsami.8b22021. 30758181

[26] S. Zhang , J. Zhu , Y. Zhang , et al., “Standalone Stretchable RF Systems Based on Asymmetric 3D Microstrip Antennas With on‐Body Wireless Communication and Energy Harvesting,” Nano Energy 96 (2022): 107069.

[27] B.‐Y. Yu , D.‐W. Yue , K.‐X. Hou , et al., “Stretchable and Self‐Healable Spoof Plasmonic Meta‐Waveguide for Wearable Wireless Communication System,” Light: Science & Applications 11 (2022): 307, 10.1038/s41377-022-01005-1. PMC9592613 36280662

[28] S. H. Kim , A. Basir , R. Avila , et al., “Strain‐Invariant Stretchable Radio‐Frequency Electronics,” Nature 629 (2024): 1047–1054, 10.1038/s41586-024-07383-3. 38778108

[29] X. Tian , P. M. Lee , Y. J. Tan , et al., “Wireless Body Sensor Networks Based on Metamaterial Textiles,” Nature Electronics 2 (2019): 243–251, 10.1038/s41928-019-0257-7.

[30] X. Tian , Q. Zeng , S. A. Kurt , et al., “Implant‐to‐Implant Wireless Networking With Metamaterial Textiles,” Nature Communications 14 (2023): 4335, 10.1038/s41467-023-39850-2. PMC10356940 37468458

[31] X. Zhu , K. Wu , X. Xie , S. Anderson , and X. Zhang , “A Robust Near‐Field Body Area Network Based on Coaxially‐Shielded Textile Metamaterial,” Nature Communications 15 (2024): 6589, 10.1038/s41467-024-51061-x. PMC11297955 39097604

[32] R. Lin , H. Kim , S. Achavananthadith , et al., “Wireless Battery‐Free Body Sensor Networks Using Near‐Field‐Enabled Clothing,” Nature Communications 11 (2020): 444. 10.1038/s41467-020-14311-2 PMC6978350 31974376

[33] Z. Kou , C. Zhang , B. Yu , et al., “Wearable All‐Fabric Hybrid Energy Harvester to Simultaneously Harvest Radiofrequency and Triboelectric Energy,” Advanced Science 11 (2024): 2309050, 10.1002/advs.202309050. 38380554 PMC11077651

[34] H. Chen , M.‐Y. Geng , Z.‐M. Chen , et al., “Digitally Controlled Tunable Fabric Microwave Filter Based on Organic Electrochemical Transistors,” Advanced Materials Technologies 8 (2023): 2300428, 10.1002/admt.202300428.

[35] K. Zhang , L. Zheng , F. I. Farha , L. Wang , and F. Xu , “Three‐Dimensional Textile Structural Conical Conformal Microstrip Antennas for Multifunctional Flexible Electronics,” ACS Applied Electronic Materials 2 (2020): 1440–1448, 10.1021/acsaelm.0c00200.

[36] D. Vital , S. Bhardwaj , and J. Volakis , “Textile‐Based Large Area RF‐Power Harvesting System for Wearable Applications,” IEEE Transactions on Antennas and Propagation 68 (2020): 2323–2331, 10.1109/TAP.2019.2948521.

[37] J. Lee , P. Dzagbletey , M. Jang , J. Chung , and J. So , “Flat Yarn Fabric Substrates for Screen‐Printed Conductive Textiles,” Advanced Engineering Materials 22 (2020): 2000722, 10.1002/adem.202000722.

[38] H. Hong , J. Hu , and X. Yan , “UV Curable Conductive Ink for the Fabrication of Textile‐Based Conductive Circuits and Wearable UHF RFID Tags,” ACS Applied Materials & Interfaces 11 (2019): 27318–27326, 10.1021/acsami.9b06432. 31284718

[39] X. Peng , X. Meng , B. Yu , et al., “Graphitized and Flexible Porous Textile Updated From Waste Cotton for Wearable Electromagnetic Interference Shielding,” Carbon 207 (2023): 144–153, 10.1016/j.carbon.2023.02.044.

[40] S. Chen , S. Fan , J. Qi , et al., “Ultrahigh Strain‐Insensitive Integrated Hybrid Electronics Using Highly Stretchable Bilayer Liquid Metal Based Conductor,” Advanced Materials 35 (2023): 2208569. 10.1002/adma.202208569 36353902

[41] C. Gu , W. Qin , X. Guo , et al., “Highly Conductive, Super‐Stretchable and Stable Stretchable Conductor With CNT and Liquid Metal Alternating Layered Structure,” Chemical Engineering Journal 487 (2024): 150589, 10.1016/j.cej.2024.150589.

[42] C. Dong , A. Leber , T. Das Gupta , et al., “High‐Efficiency Super‐Elastic Liquid Metal Based Triboelectric Fibers and Textiles,” Nature Communications 11 (2020): 3537, 10.1038/s41467-020-17345-8. PMC7363815 32669555

[43] M. Kubo , X. Li , C. Kim , et al., “Stretchable Microfluidic Radiofrequency Antennas,” Advanced Materials 22 (2010): 2749–2752. 20414886 10.1002/adma.200904201

[44] G. Hayes , J. So , A. Qusba , M. Dickey , and G. Lazzi , “Flexible Liquid Metal Alloy (Egain) Microstrip Patch Antenna,” IEEE Transactions on Antennas and Propagation 60 (2012): 2151–2156, 10.1109/TAP.2012.2189698.

[45] K. Yamagishi , W. Zhou , T. Ching , S. Huang , and M. Hashimoto , “Ultra‐Deformable and Tissue‐Adhesive Liquid Metal Antennas With High Wireless Powering Efficiency,” Advanced Materials 33 (2021): 2008062. 10.1002/adma.202008062 34031936

[46] Y. Wu , S. Alkaraki , S. Tang , Y. Wang , and J. Kelly , “Circuits and Antennas Incorporating Gallium‐Based Liquid Metal,” Proceedings of THE IEEE 111 (2023): 955–977, 10.1109/JPROC.2023.3285400.

[47] R. Lin , H.‐J. Kim , S. Achavananthadith , et al., “Digitally‐Embroidered Liquid Metal Electronic Textiles for Wearable Wireless Systems,” Nature Communications 13 (2022): 2190, 10.1038/s41467-022-29859-4. PMC9023486 35449159

[48] W. Zhu , X. Liu , Y. Fan , Z. Zhang , and X. Ma , “Transparent and Flexible Antenna With Polarization Reconfigurability Using EgaIn for Wearable Applications,” IEEE Transactions on Antennas and Propagation 72 (2024): 7493–7503, 10.1109/TAP.2024.3439882.

[49] K. Yamagishi , T. Ching , and N. Chian , “Flexible and Stretchable Liquid‐Metal Microfluidic Electronics Using Directly Printed 3D Microchannel Networks,” Advanced Functional Materials 34 (2024): 2311219, 10.1002/adfm.202311219.

[50] Q. Zhuang , K. Yao , C. Zhang , et al., “Permeable, Three‐Dimensional Integrated Electronic Skins With Stretchable Hybrid Liquid Metal Solders,” Nature Electronics 7 (2024): 598–609, 10.1038/s41928-024-01189-x.

[51] Y. Lu , D. Yu , H. Dong , et al., “Dynamic Leakage‐Free Liquid Metals,” Advanced Functional Materials 33 (2023): 2210961, 10.1002/adfm.202210961.

[52] G. Park , G. Lee , W. Lee , J. Kang , S. Park , and S. Park , “Divide and Conquer: Design of Gallium‐Based Liquid Metal Particles for Soft and Stretchable Electronics,” Advanced Functional Materials 34 (2024): 2309660, 10.1002/adfm.202309660.

[53] Q. Wei , M. Sun , Z. Wang , et al., “Surface Engineering of Liquid Metal Nanodroplets by Attachable Diblock Copolymers,” ACS Nano 14 (2020): 9884–9893, 10.1021/acsnano.0c02720. 32649179

[54] Y. Lin , J. Genzer , and M. D. Dickey , “Attributes, Fabrication, and Applications of Gallium‐Based Liquid Metal Particles,” Advanced Science 7 (2020): 2000192, 10.1002/advs.202000192. 32596120 PMC7312306

[55] L. Yang , Z. Wang , H. Wang , et al., “Self‐Healing, Reconfigurable, Thermal‐Switching, Transformative Electronics for Health Monitoring,” Advanced Materials 35 (2023): 2207742, 10.1002/adma.202207742. PMC10391699 36719993

