# Supplementary material for: Printable Liquid Metal‐Textiles for Deformation‐Insensitive and Electromagnetically Robust mmWave Devices
Source: Adv Sci (Weinh). 2026 Mar 19;13(28):e22350. doi: 10.1002/advs.202522350 (PMC13185834; doi:10.1002/advs.202522350)
Supplement: Supplementary file 1 — Supporting File 1: advs74739‐sup‐0001‐SuppMat.docx. [file ADVS-13-e22350-s002.docx]

Supporting Information

Printable Liquid Metal-Textiles for Deformation-Insensitive and Electromagnetically Robust mmWave Devices

Lu Ju, Buyun Yu, Rui Wang, Hao Chen, Kexin Hou, Chao Zhang, Mingyang Geng, Yunqian Dai, Yingying Zhang, Ying-Shi Guan^*^, Cunjiang Yu^*^, and Wei-Bing Lu^*^

L. Ju, W.-B. Lu

School of Information and Intelligent Science, Donghua University, Shanghai 201620, China

E-mail: [wblu@seu.edu.cn](mailto:wblu@seu.edu.cn)

L. Ju, B. Yu, H. Chen, C. Zhang, M. Y. Geng, W.-B. Lu

State Key Laboratory of Millimeter Waves, School of Information Science and Engineering, Southeast University, Nanjing 210096, China

L. Ju, B. Yu, H. Chen, M. Y. Geng, W.-B. Lu

Center for Flexible RF Technology, Frontiers Science Center for Mobile Information Communication and Security, Southeast University, Nanjing 210096, China

R. Wang, Y. Q. Dai, Y.-S. Guan

School of Chemistry and Chemical Engineering, Southeast University, Nanjing 211189, China

E-mail: [gyshi412@seu.edu.cn](mailto:gyshi412@seu.edu.cn)

K. Hou

State Key Laboratory of Coordination Chemistry, School of Chemistry and Chemical Engineering, Collaborative Innovation Center of Advanced Microstructures, Nanjing University, Nanjing 210023, China

Y. Zhang

Key Laboratory of Organic Optoelectronics and Molecular Engineering of the Ministry of Education, Department of Chemistry, Tsinghua University, Beijing, 100084 China

C. Yu

Department of Electrical and Computer Engineering, Department of Bioengineering, Departments of Materials Science and Engineering, Department of Mechanical Science and Engineering, Beckman Institute for Advanced Science and Technology, Materials Research Laboratory, Nick Holonyak Micro and Nanotechnology Laboratory, University of Illinois, Urbana-Champaign; Urbana, IL 61801, USA

E-mail: [cunjiang@illinois.edu](mailto:cunjiang@illinois.edu)

L. Ju, B. Yu contributed equally to this work

Keywords: Liquid metal, Millimeter-wave devices, Electronic textiles, Deformation-insensitive, Wireless communication

**Supporting Figures**


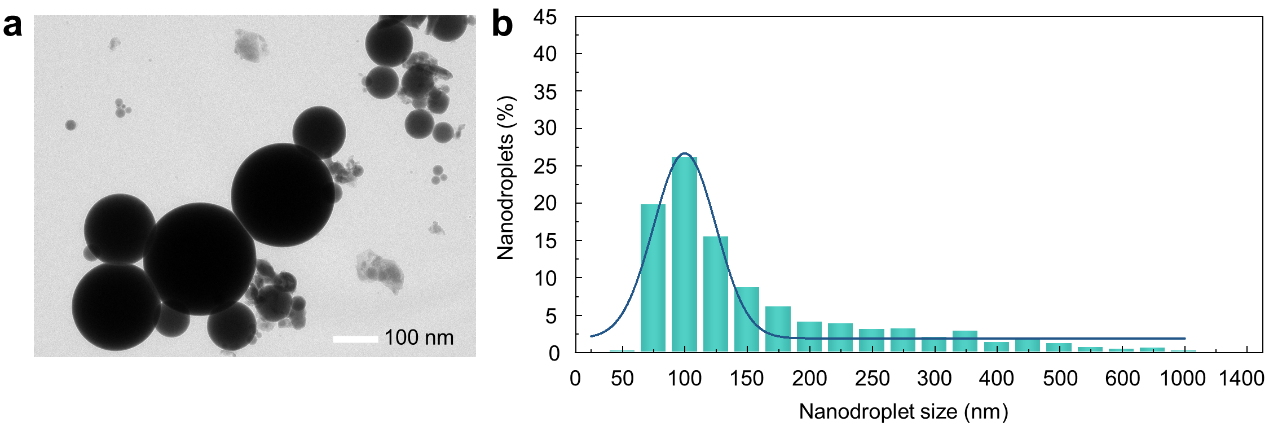


**Figure S1** Characterizations of LM nanodroplets by high-power ultrasonication. (a) TEM image, and (b) size distribution of LM nanodroplets. The gallium oxide (Ga_2_O_3_) layer was spontaneously formed on the surface of the LM nanodroplets, which reduces the surface tension and coalescence of the LM nanodroplets. The fractured spherical LM nanodroplets exhibit a diameter of approximately 100 nm.


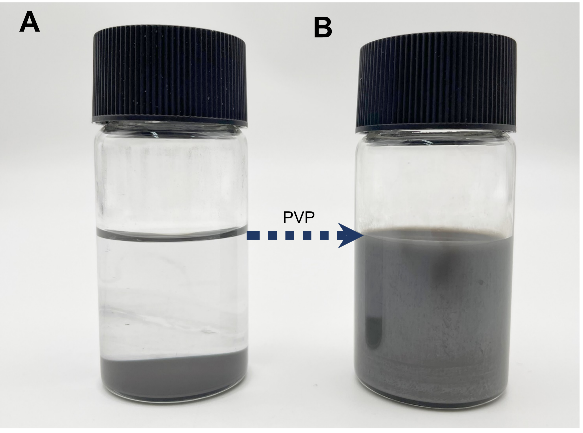


**Figure S2** Stability of the LM nanodroplets in (a) isopropanol solvent and (b) PVP stabilizer. Without the modification of PVP nanolayer, LM nanodroplets in isopropanol solvent exhibit significant aggregation and precipitation after 0.5 days. On the contrary, PVP-stabilized LM nanodroplets maintain good dispersion and exhibit sustained colloidal stability in isopropanol over 7 days.


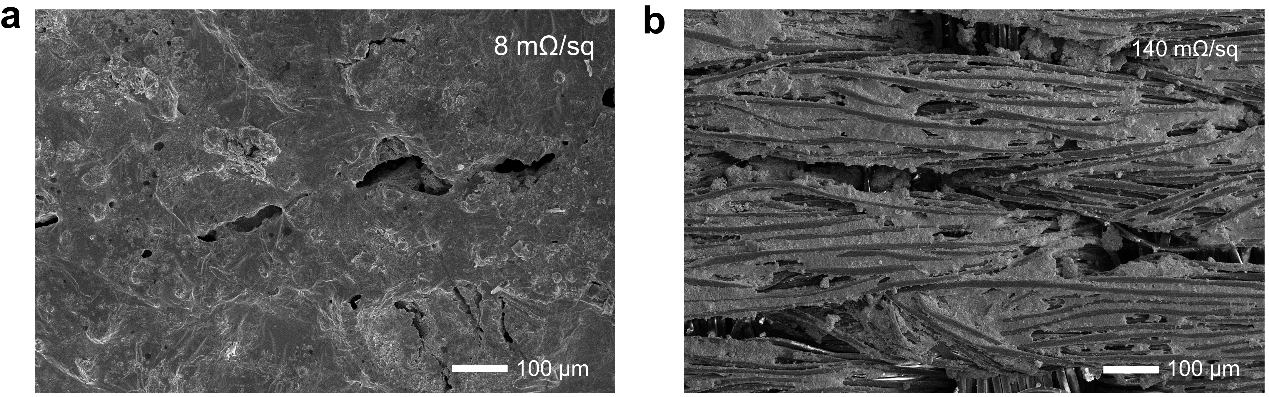


**Figure S3** SEM images of the LM-textiles. The surface morphology of the (a) stencil printed and (b) screen-printed LM-textiles.


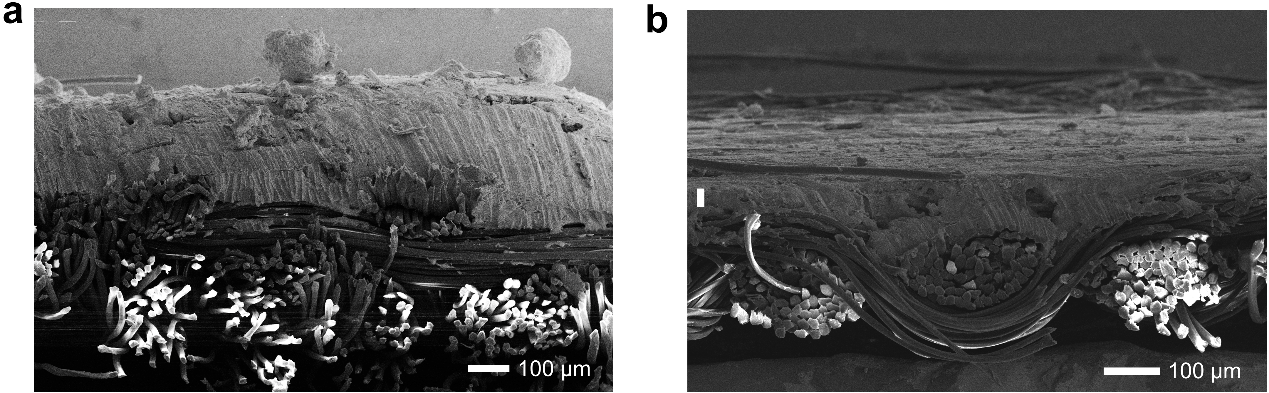


**Figure S4** SEM images of the LM-textiles. The cross-section of the (a) stencil printed and (b) screen-printed LM-textiles. SEM images reveal a significant difference in the thicknesses of the conductive layers fabricated via stencil printing (~200 μm) and screen printing (~33 μm).


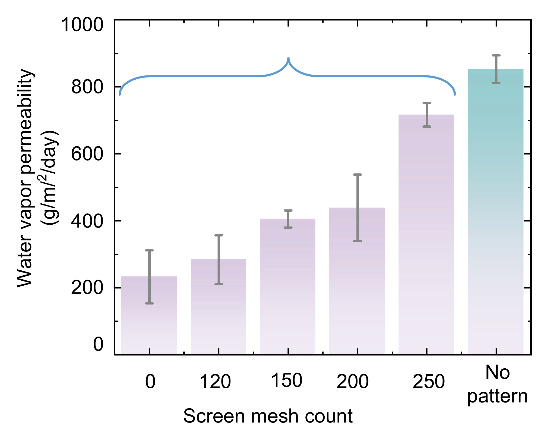


**Figure S5** Water vapor permeability of the LM-textiles based on “single-mask” printing, “dual-mask” printing with 120, 150, 200, 250 screen mesh count, and blank textile without printing functionalized LM inks. The water vapor permeability test was performed at 25 °C and 30% relative humidity (testing duration: 72 h). It can be observed that the water vapor transmission rate of the LM-textiles decreases as the mesh count increases. Data presented as mean ± SEM, n=3.


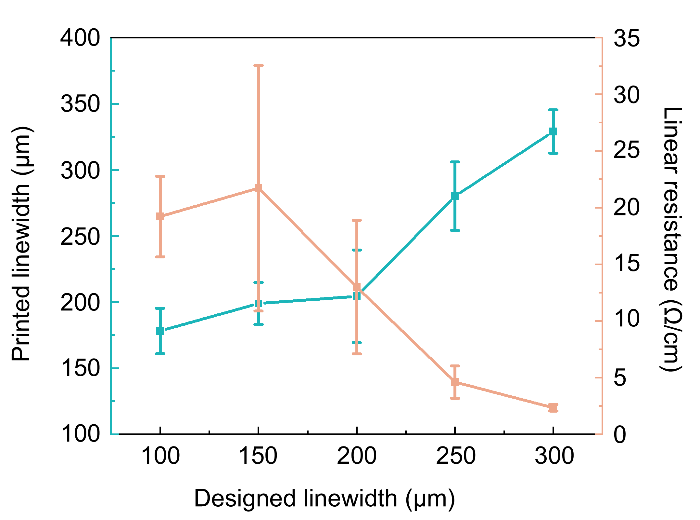


**Figure S6** Statistical distributions of printed linewidth resolution and linear resistance of the LM-textiles as a function of the patterning resolution. The result shows the “dual mask” printing can attain a minimum linewidth of 150 (±58.2) µm and an average electrical performance of 21.72 Ω/cm in textiles. Data presented as mean ± SEM, n=6.


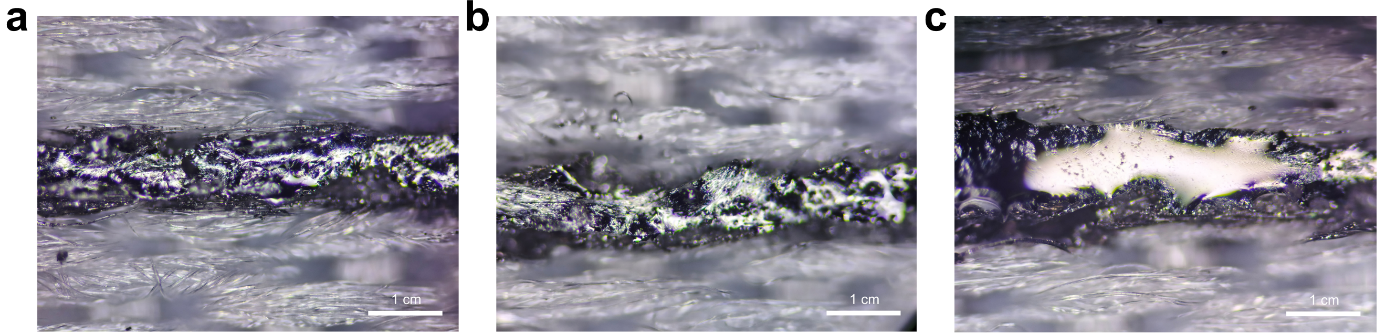
**Figure S7** Optical images of the LM-textiles fabricated by (a) “dual mask” printing with a 120-mesh screen, (b) “dual mask” printing with a 250-mesh screen, (c) “single-mask” stencil printing at a magnification of 100X. Figure S7a shows a well-defined and uniform LM inks coating in textile. With increasing screen density, the limited penetration of functionalized nanodroplets results in non-uniform or broken lines on the textile substrate (Figure S7b).


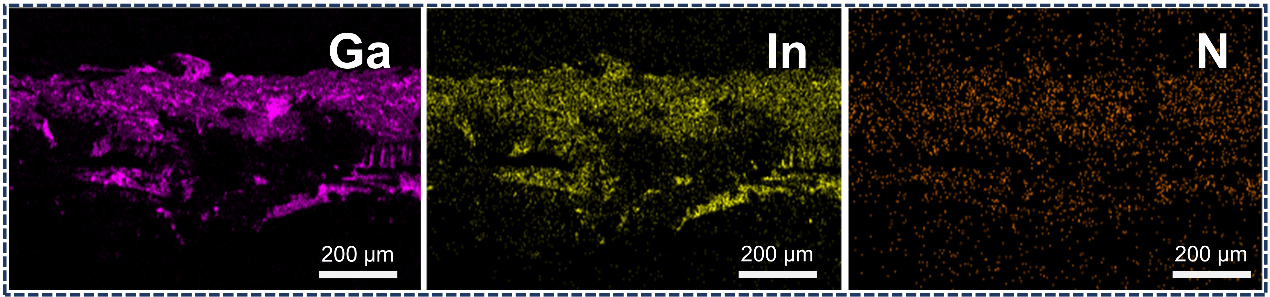


**Figure S8** The corresponding EDS analysis of the printed LM-textile circuits. The related EDS images show that the N is evenly distributed in the LM-textile matrix, which indicates that the surface of the Ga_2_O_3_ nanolayers is covered by PVP nanolayers. In addition, the uniform distribution of elements indicates that functionalized LM nanodroplets exhibit strong adhesion to the textile surface when subjected repeated mechanical bending.


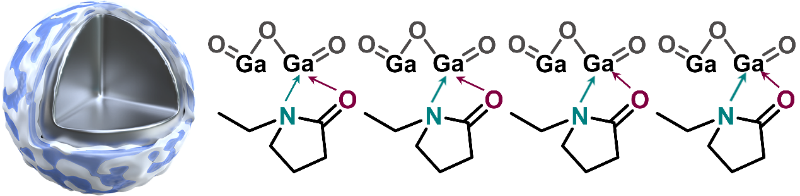


**Figure S9** The formation of PVP-modified LM nanodroplets.


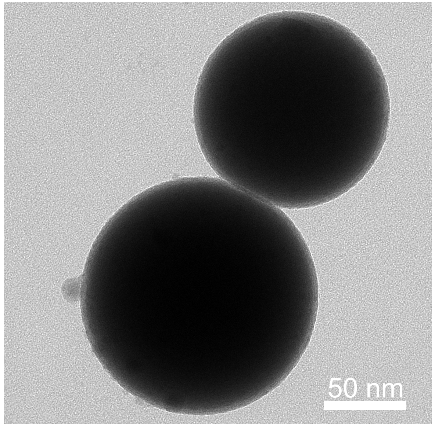


**Figure S10** TEM image of LM nanodroplets without PVP modification.


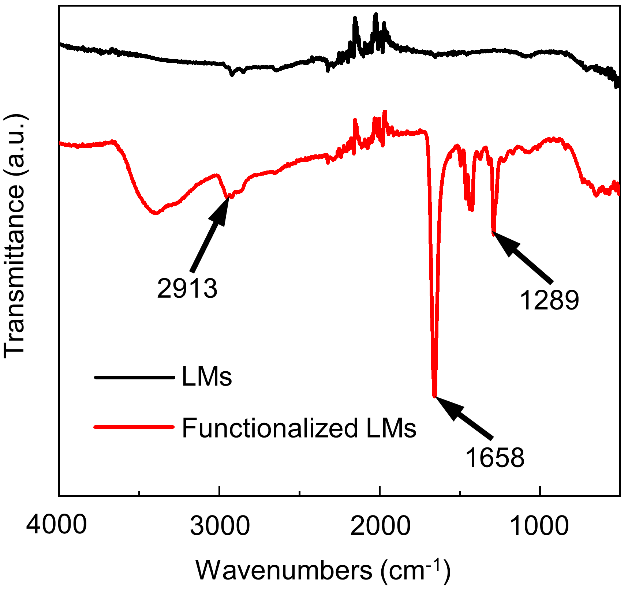


**Figure S11** FTIR spectra of large pristine LM droplets (LMs) and functionalized LM nanodroplets (Functionalized LMs). It is evident that there is no absorption peak in the FTIR spectra of large pristine LMs. On the contrary, the functionalized LMs exhibit significant absorption peaks at characteristic positions of 2913 cm^-1^, 1658 cm^-1^, and 1289 cm^-1^. The three absorption peaks correspond to the peaks of C-H, C=O, and C-N stretching vibration, respectively. These characteristic absorption peaks strongly prove PVP modification on the surface of LMs.


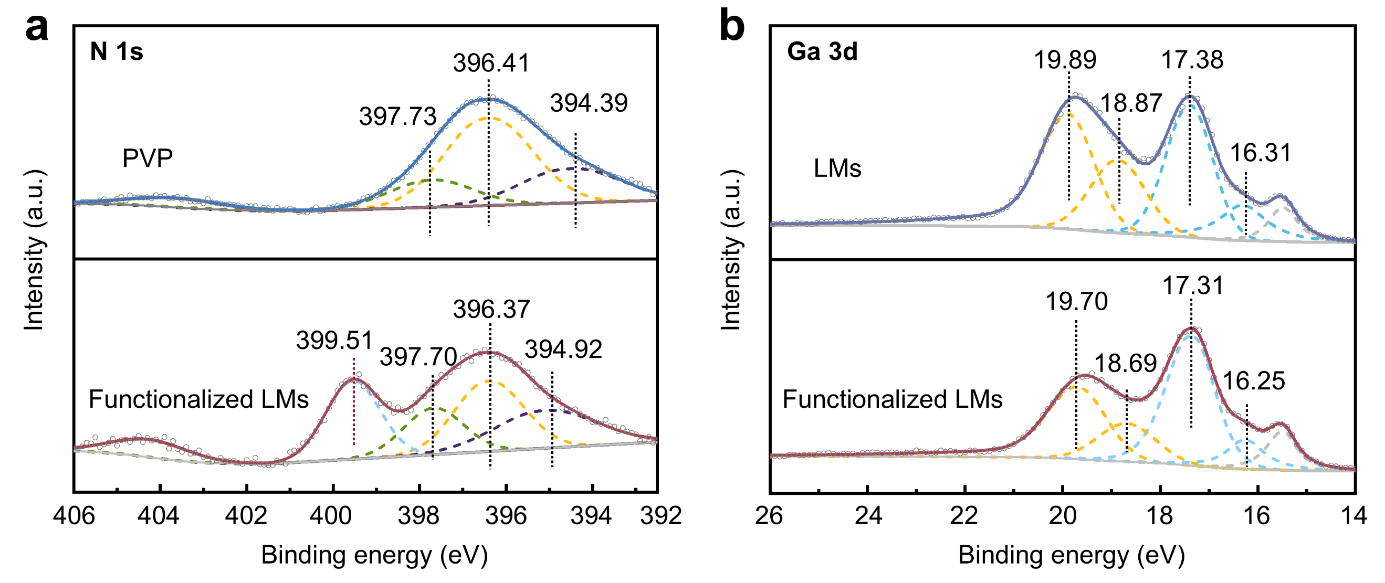


**Figure S12** XPS spectra of (a) N 1s of PVP and functionalized LM nanodroplets (Functionalized LMs). (b) Ga 3d of LM nanodroplets without PVP (LMs) and functionalized LMs. The N 1s spectrum of PVP exhibits three components at 394.39, 396.41, and 397.73 eV, while the Ga 3d spectrum of LM nanodroplets without PVP can be deconvoluted into four peaks located at 16.31, 17.38, 18.87, and 19.89 eV. After the introduction of PVP, a new N 1s component emerges at 399.84 eV. Meanwhile, the N 1s component originally located at 394.39 eV shifts toward higher binding energy, indicating a decrease in the local electron density around the N atoms. In contrast, the Ga 3d peaks exhibit an overall shift toward lower binding energy after PVP functionalization.These observed XPS shifts suggest the formation of coordination interaction between PVP and LMs.


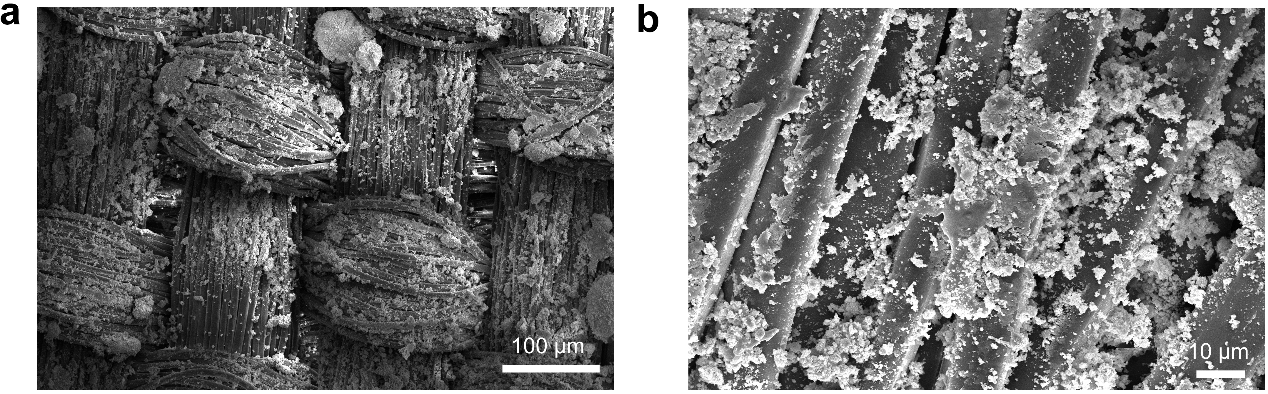


**Figure S13** SEM images of LM nanodroplets without surface modification of PVP printed on (a) textiles (b) single fiber. We can clearly observe the unmodified LM nanodroplets are sparsely distributed over the textile substrate. Significantly, the textile is almost non-conductive.


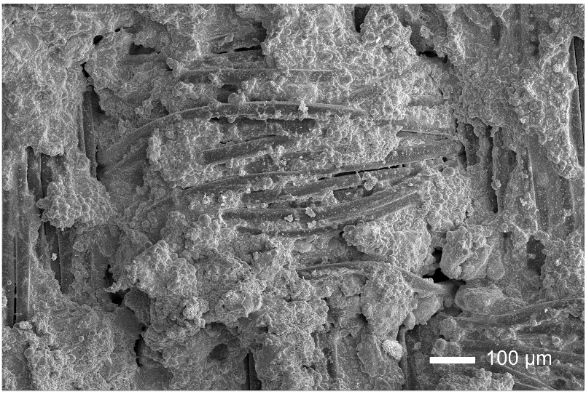


**Figure S14** SEM images of LM nanodroplets with low concentration of PVP printed on LM-textiles. As revealed by the SEM image, the surface modification of PVP allows more functionalized LM nanodroplets attached to textile substrate. However, the low concentration of PVP cannot guarantee uniform encapsulation of LM nanodroplets, resulting in aggregation of nanodroplets and discontinuous conductive network.


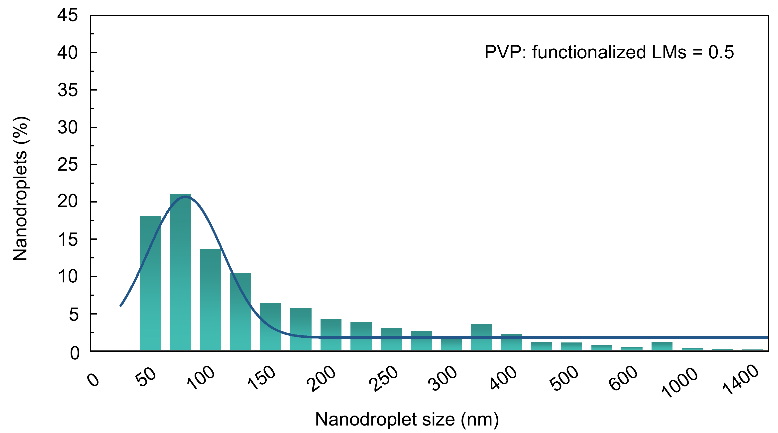


**Figure S15** Size distribution of LM nanodroplets with low concentration of PVP. The mass ratio of PVP to functionalized LM nanodroplets is 0.5. The broader size distribution indicates agglomeration at low concentrations due to insufficient surface modification.


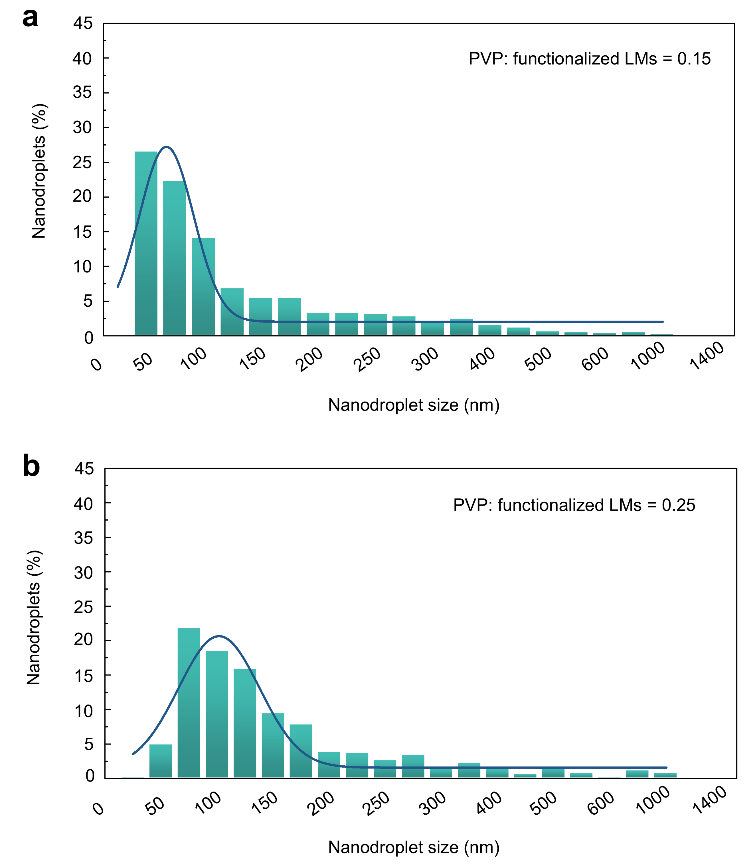


**Figure S16** Size distribution of LM nanodroplets with high concentration of PVP. The mass ratios of PVP to functionalized LM nanodroplets are 0.15 and 0.25, respectively. As the PVP concentration increase, the size distributions become broader and some larger agglomerates occur.


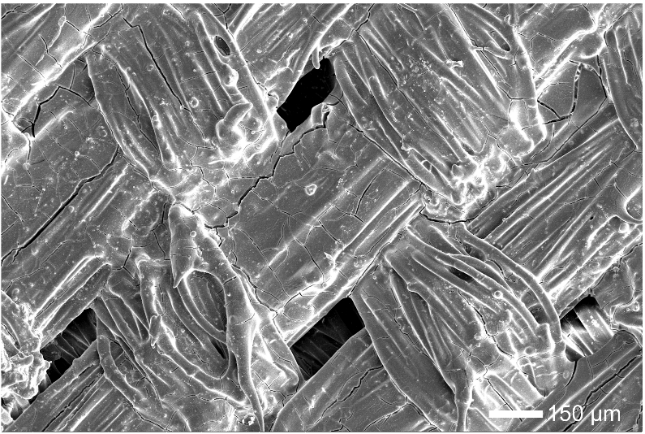


**Figure S17** SEM images of LM-textiles. It is significantly noted that the surface morphology of LM-textiles became smoother and some big cracks appeared in the conductive path at high PVP concentration.


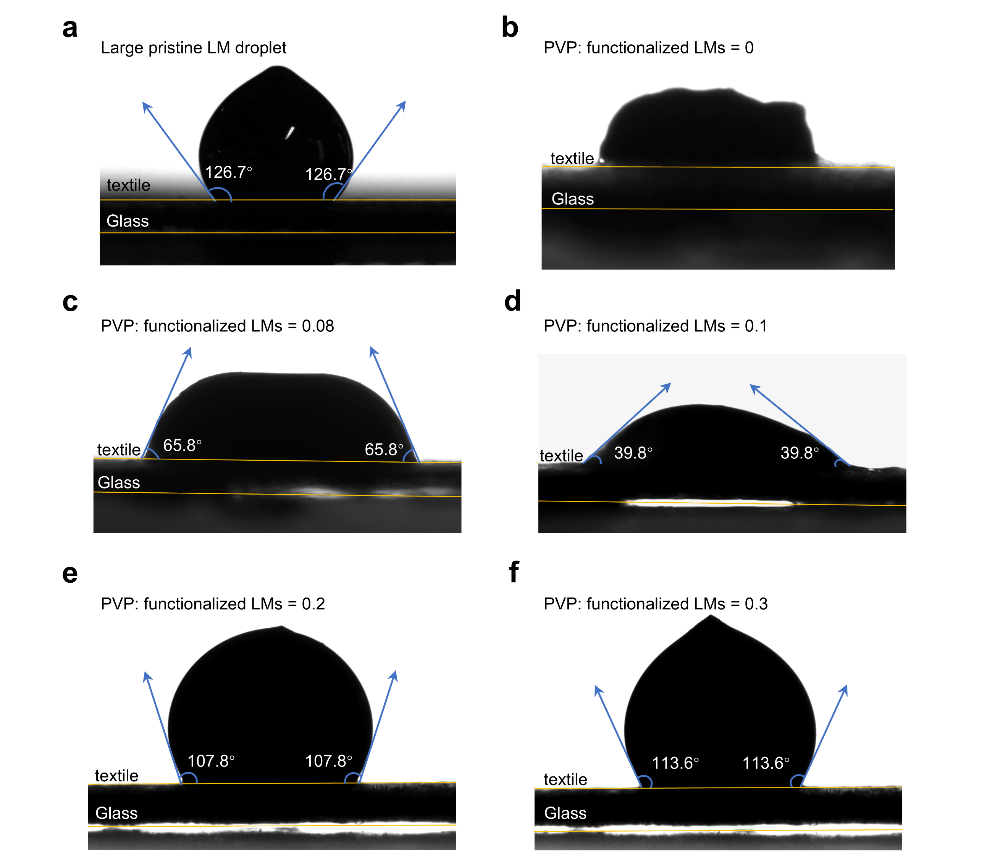


**Figure S18** Contact angles measurement. Contact angle of (a) large pristine LM droplet, (b) LM nanodroplet without modification of PVP, and (c-f) functionalized LM nanodroplets under different concentrations of PVP. Functionalized LM nanodroplets and PVP as a ratio of (c) 1: 0.08; (d) 1: 0.1; (e) 1: 0.2; (f) 1:0.3. It is evident that PVP nanolayers improve the compatibility between LM nanodroplets and textiles. In addition, the contact angle will increase due to the excessive PVP.


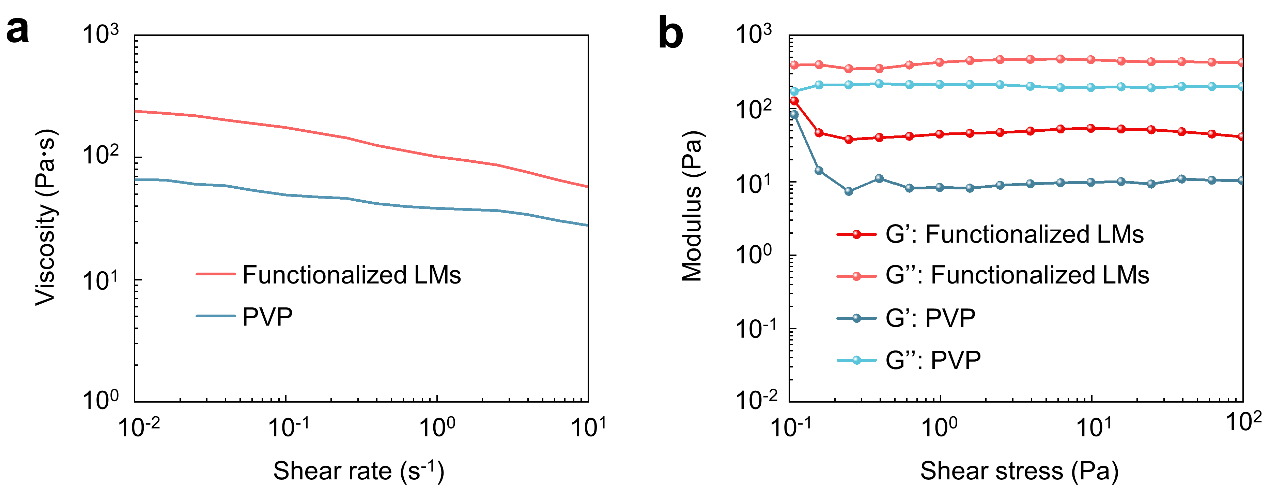


**Figure S19** Rheological properties characterization of PVP solution and LM funcitionalized LMs with excessive PVP. (a) The viscosity versus shear rate, and (b) storage and loss moduli of the functionalized LM nanodroplets (functionalized LMs) and PVP solution. The mass ratio of PVP to functionalized LMs is 0.3. The viscosity of functionalized LMs decreases when excessive PVP is added. It is noted that the PVP plays a dominant role in rheological properties of functionalized LMs when the concentrations of PVP exceeds the appropriate ratio.


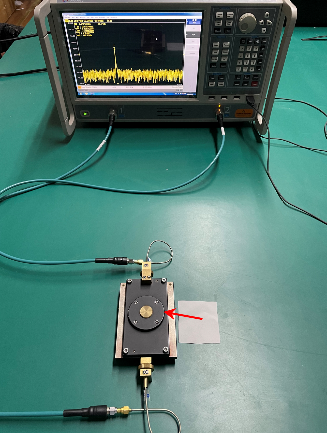


**Figure S20** Dielectric characteristic measurement. The dielectric properties of the textile substrate were obtained by a split post dielectric resonator (QWED F-SPDR-5.1). The vector network analyzer (model: Transcom T5260C) was used to measure the resonant frequency and Q factor of the split post dielectric resonator. As the textile substrate is inserted into the resonator cavity, the resonant frequency and Q factor will be changed. We calculate the dielectric constant of the textile substrate by comparing the shift in resonant frequency in the resonant frequencies between the empty resonator and the resonator loaded with the textile substrate. The dielectric loss of the textile substrate is calculated by the change in the Q factors caused by inserting the textile substrate.


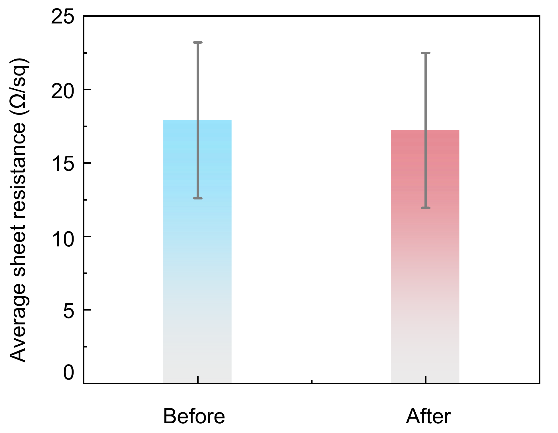


**Figure S21** Measure average sheet resistance of the LM-textile before and after peeling of the heat-release tape. We conducted electrical measurements at multiple sites across the LM-textile (80 mm ×15 mm) to assess its conductivity. The LM-textile shows an average sheet resistance of 17.91 mΩ/sq before peeling of the heat-release tape. After peeling of the heat-release tape, the average sheet resistance of the LM-textile is 17.22 mΩ/sq. The results demonstrate that the LM-textile maintains stable electrical performance after peeling of the heat-release tape. Data presented as mean ± SEM, n=10.


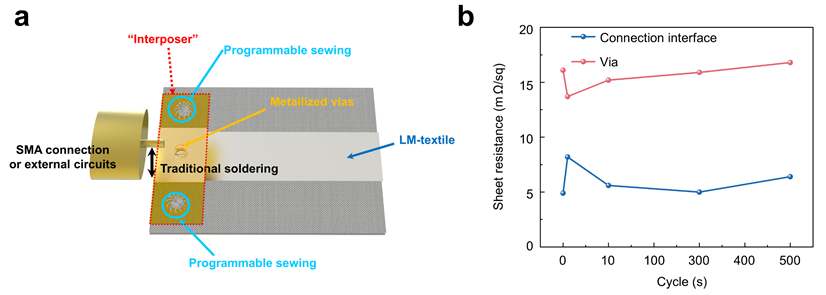


Figure S22 The reliable connection interface between LM-textiles and external circuits. (a) The connection procedure between LM-textile and SMA or external circuits. (b) The measured sheet resistance of the connection interface and via as a function of 500 manual bending cycles. The interposer is a thin and flexible copper-clad PI with metallized vias, which is compatible with conventional high-temperature soldering processes. We integrate connector or external circuits onto the interposer via soldering. To further ensure reliable interconnections between the interposer and LM-textile, we take programmable sewing method and infuse silver paste into the vias. We conducted strain tests on connection region and measured the electrical performance at both the connection interface and the metallized via. After repeated deformation, both the connection interface and the metallized via maintain mechanical integrity. Their sheet resistances are 6.4 mΩ/sq and 16.8 mΩ/sq after 500 bending cycles, respectively.


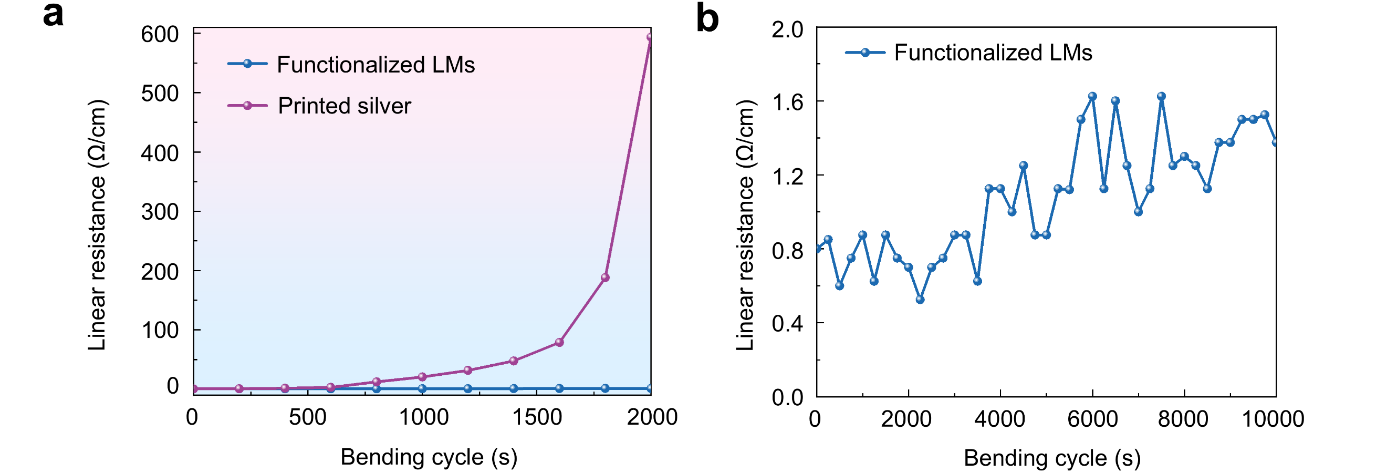


**Figure S23** Electrical characterization. Linear resistances of (a) the LM-textile and printed silver-textile as a function of 2000 bending cycles, (b) the LM-textile as a function of 10,000 bending cycles. The bending radius and rate are set as 7.5 mm, 5 mm/s, respectively. The LM-textile demonstrates excellent stability in electrical conductivity under repeated bending, in contrast to the degradation observed in silver-textile.


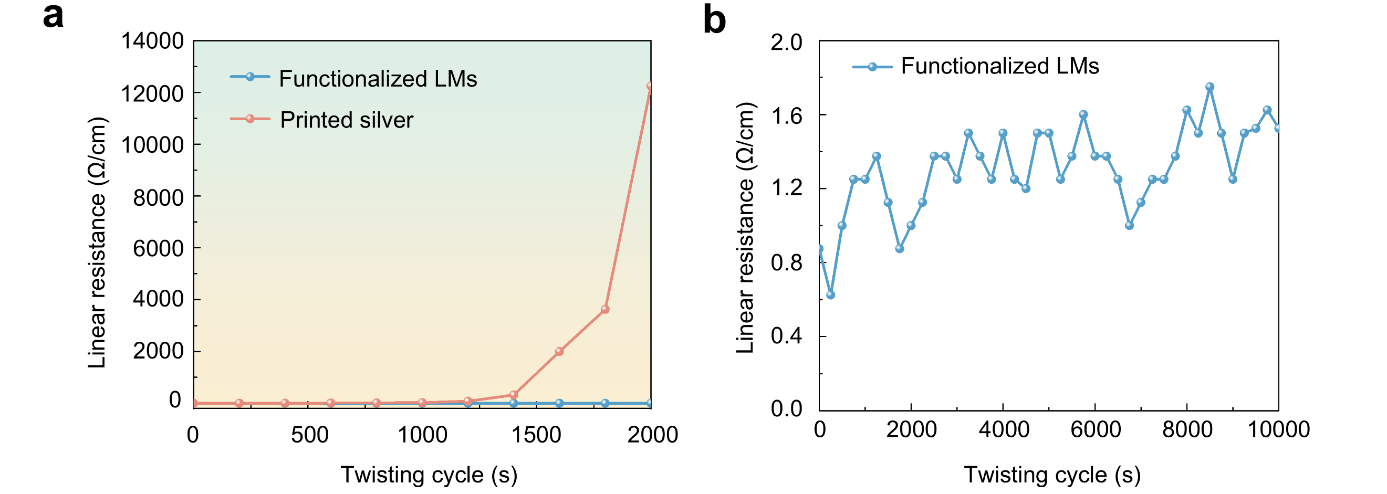


**Figure S24** Electrical characterization. Linear resistances of (a) the LM-textile and printed silver-textile as a function of 2000 twisting cycles, (b) the LM-textile as a function of 10,000 twisting cycles. The twisting angle was set to 180°. Mechanical stress exerts a significant influence on the resistance of silver-textile, while the LM-textile maintain stable electrical properties even after repeated cyclic twisting.


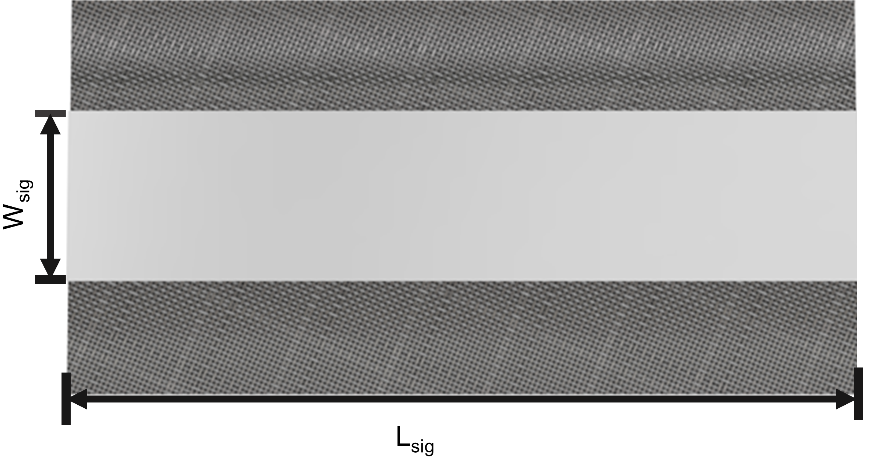


**Figure S25** The detailed geometric parameters and prototype of the fabricated LM-textile microstrip transmission line. *W_sig_*= 4.3 mm, *L_sig_*= 30 mm.


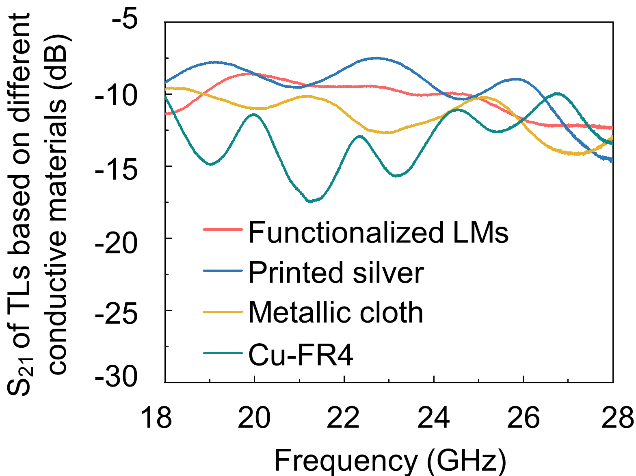


**Figure S26** S-parameter measurements. Measured insertion loss (S_21_) of microstrip transmission lines based on functionalized LM nanodroplets (functionalized LMs) and other conductive materials in mmWave frequency band.


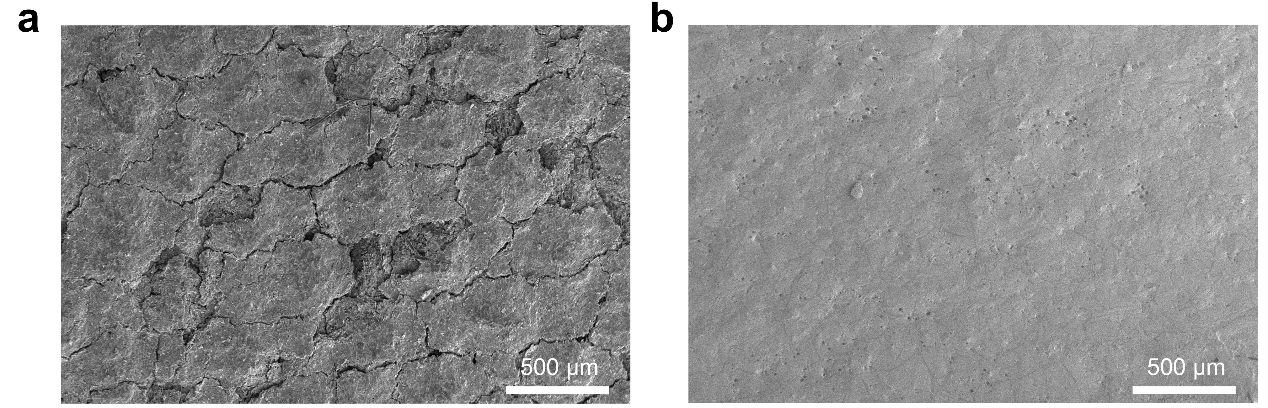


**Figure S27** SEM images of the surface morphology of the (a) printed silver-textile, and (b) printed LM-textile circuit after mechanical tests. Compared with the printed silver-textile that exhibits severe surface cracking, the LM-textile remains dense and intact conductive pathway.


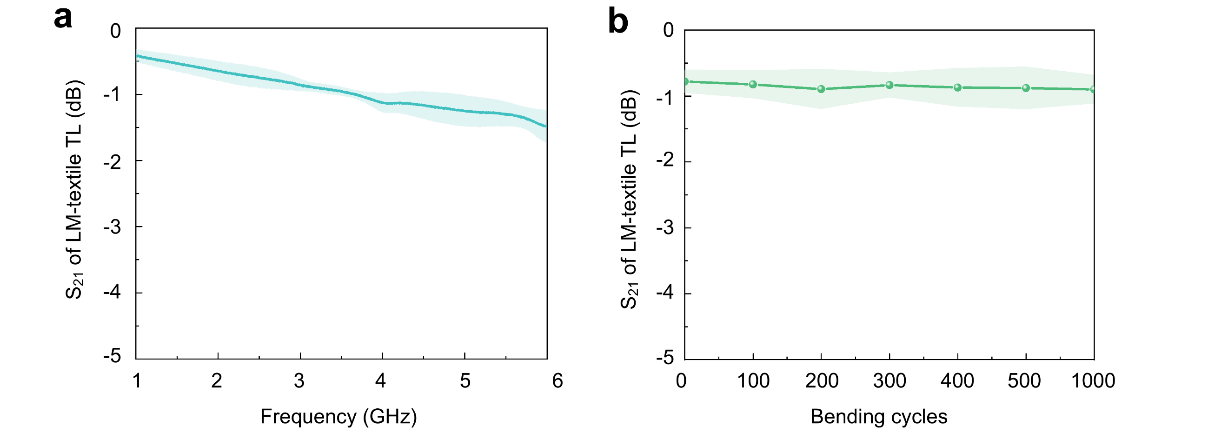


**Figure S28** S-parameter measurements at sub-6G frequency. Measured insertion loss (S_21_) of four microstrip transmission lines with same structures at (a) flat state (1-6 GHz) , and (b) bending state (2.45 GHz). Data presented as mean ± SEM, n=4.


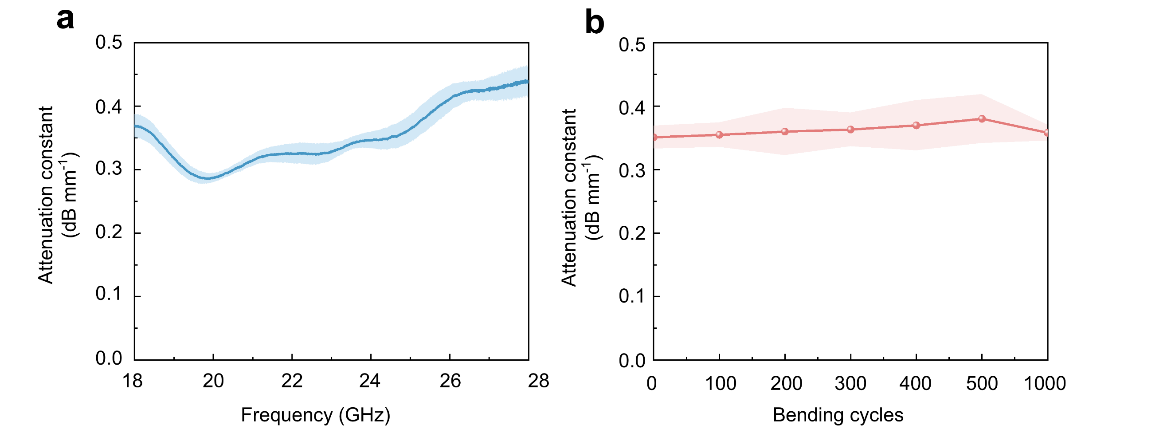


**Figure S29** Measured attenuation constant of four microstrip transmission lines with same structures at (a) flat state (18-28 GHz), and (b) bending state (24.5 GHz). The insertion loss of measured LM-textile microstrip TLs particularly at mmWave frequencies is attributed to different parts: conductor loss, substrate loss, radiation losses, connector loss, and transition losses. Data presented as mean ± SEM, n=4.


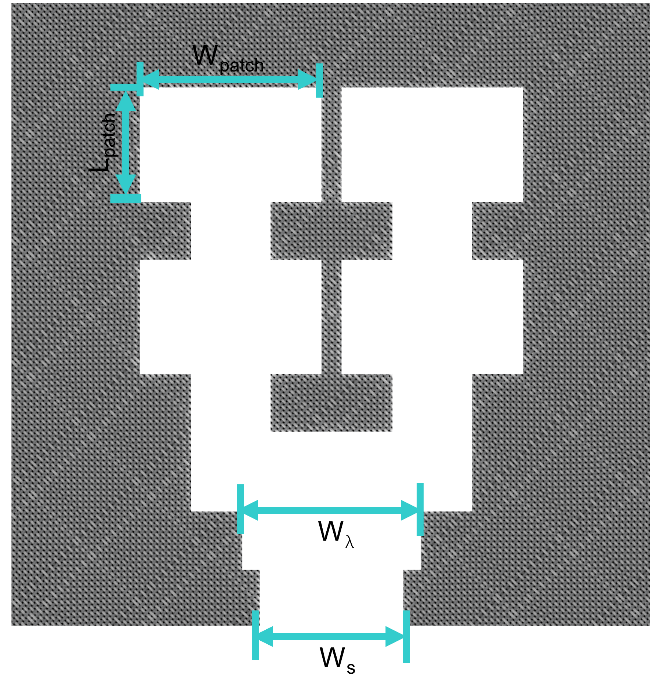


**Figure S30** The detailed geometric parameters and prototype of the fabricated 2×2 LM-textile mmWave antenna array. *W_patch_*= 5.6 mm, *L_patch_*= 3.4 mm, *W_s_*=3.9 mm, *W_λ_*= 5.8 mm.


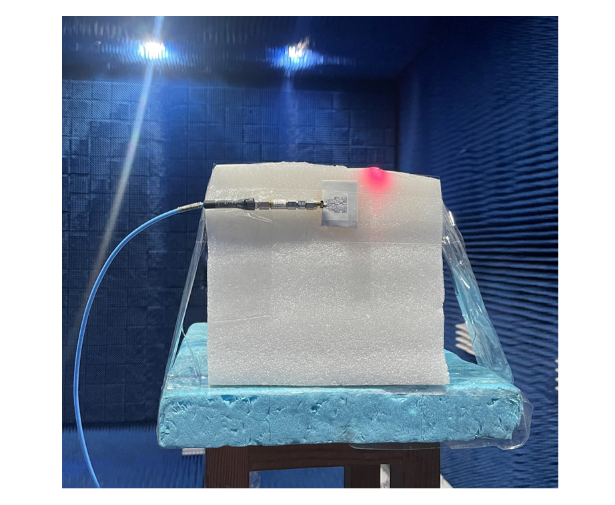


**Figure S31** Radiation pattern measurement. Photograph of radiation pattern measurement of the LM-textile mmWave antenna array in an anechoic chamber.


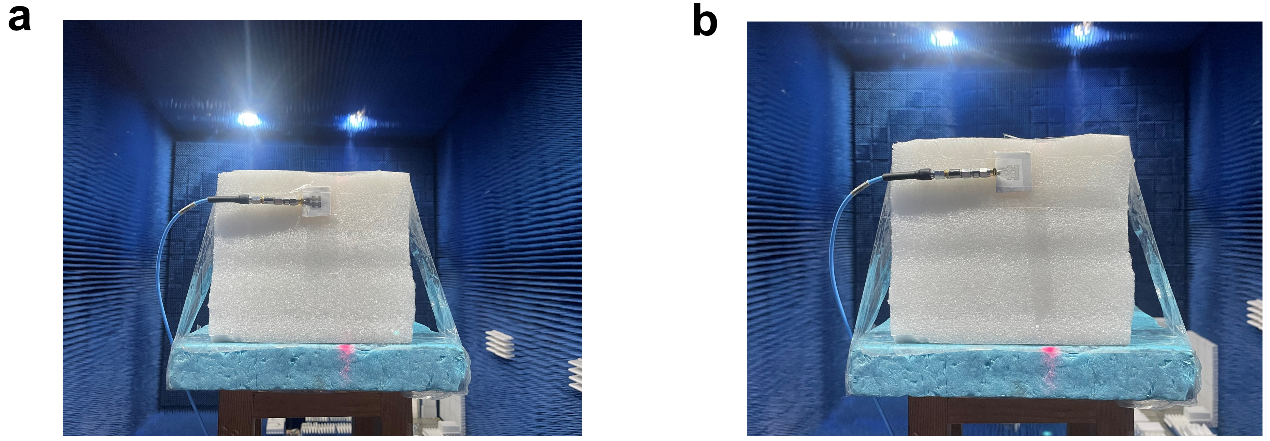


**Figure S32** Radiation pattern measurements. Photograph of radiation pattern measurements of the mmWave antenna arrays based on (a) metallic cloth and (b) printed silver inks in an anechoic chamber.


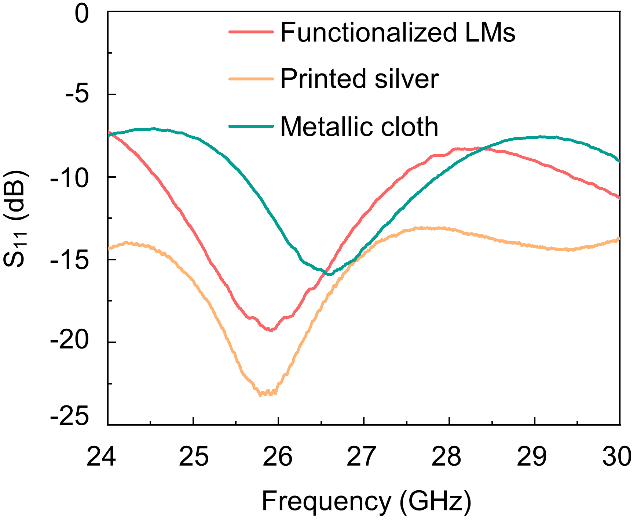


**Figure S33** S-parameter measurements. Measured return Loss (S_11_) of the 2×2 mmWave antenna arrays based on functionalized LM nanodroplets (functionalized LMs), printed silver inks, and metallic cloth. At the original state, the antenna arrays based on the three kinds of materials exhibit good impedance matching characteristics at 26 GHz.


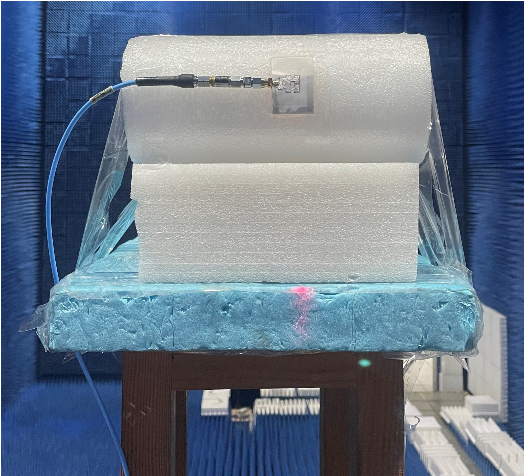


**Figure S34** Radiation pattern measurements. Photograph of radiation pattern measurement of the LM-textile mmWave antenna array conformal to a foam in an anechoic chamber.


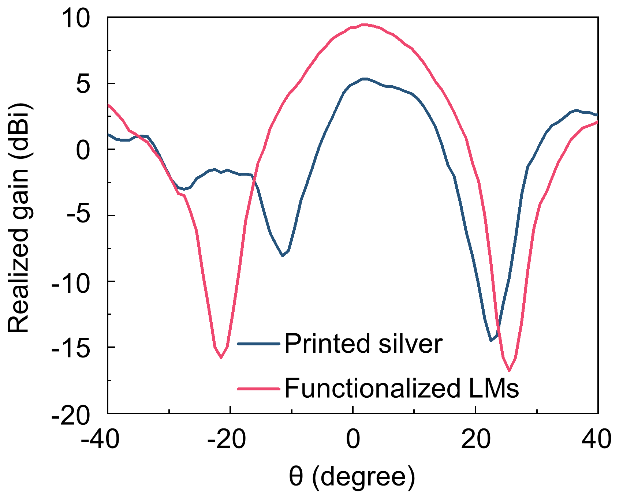


**Figure S35** Radiation pattern characterization. LM-textile antenna array retains a higher realized gain compared with printed silver-textile, indicating its superior mechanical and electromagnetic stability when subjected multiple mechanical cycles.


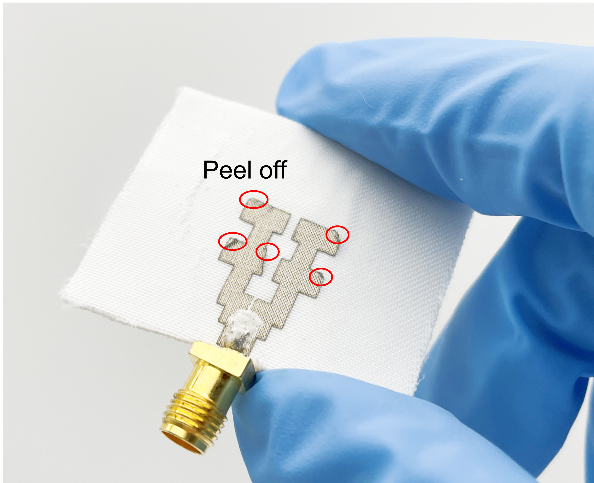


**Figure S36** Photograph of the 2×2 metallic-cloth-based mmWave antenna array after repeated bending tests. It is clearly observed that external mechanical stress severely compromises the structure of the metallic-cloth-based antenna array, resulting in partial delamination of the conductive layer from the textile substrate.


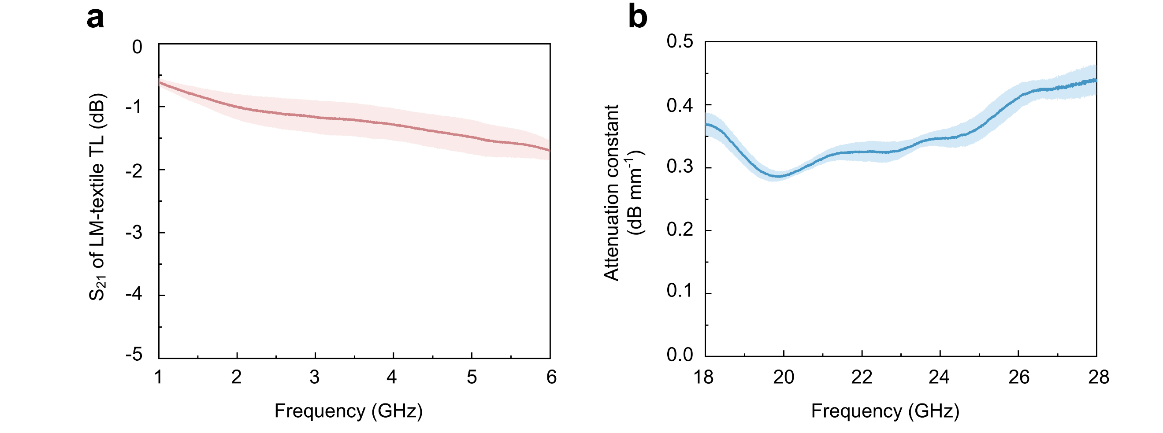


**Figure S37** Measured insertion loss of the LM-textile microstrip transmission lines after washing tests. Owing to the excellent encapsulation technique, the LM-textile microstrip transmission line shows exhibited a low average insertion loss of -1.09 dB at 2.45 GHz and an average insertion loss of 0.34189 db/mm at 24.5 GHz. Data presented as mean ± SEM, n=4.


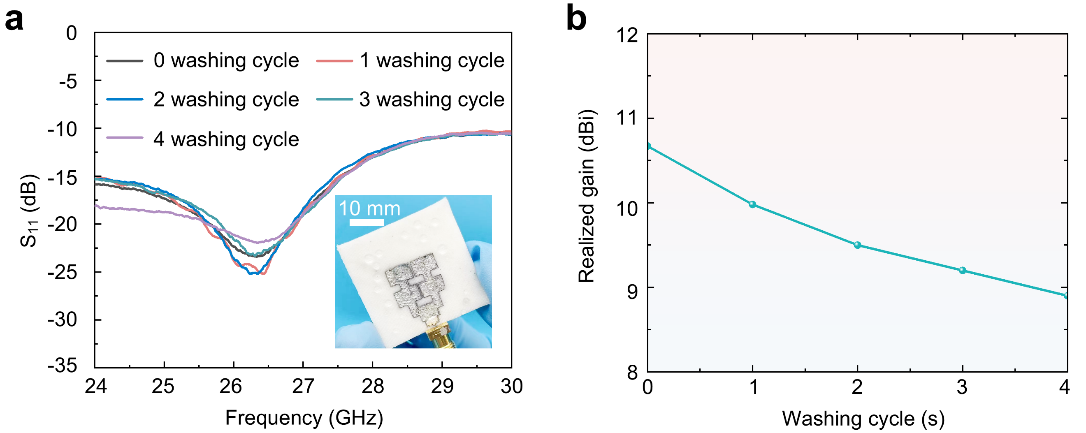


**Figure S38** Measured (a) S_11_, and (b) maximum realized gains of LM-textile mmWave antenna array after washing and friction tests. The inset photograph shows the state of the antenna after water washing tests. The experimental results indicate that this chemically stable, waterproof protective layer enables the LM-textile antenna to maintain excellent impedance matching at 26 GHz and stable radiation performance with a maximum realized gain of 8.9 dBi.


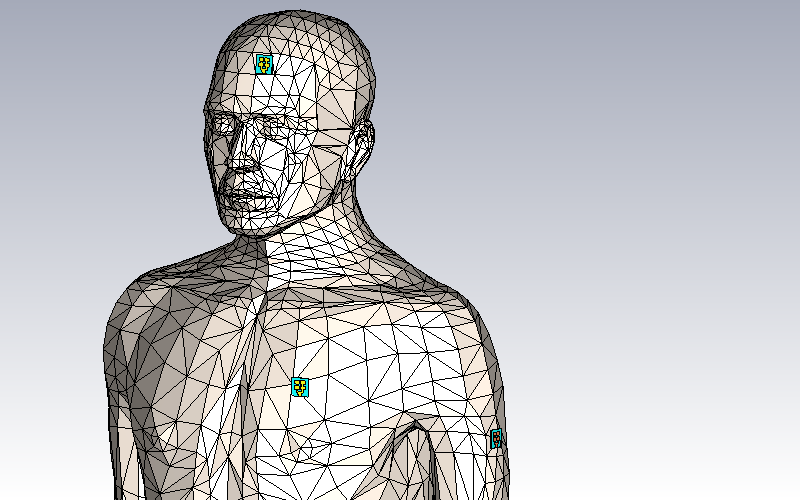


**Figure 39** The SAR simulation setup of the LM-textile mmWave antenna array.


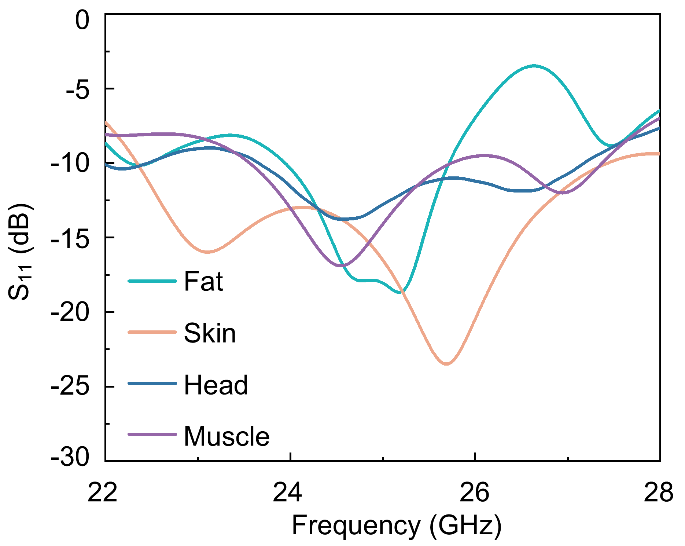


**Figure 40** Measured S_11_ of the LM-textile mmWave antenna array conformed to the various body parts of human body phantom.

Table S1 The comparison of proposed LM-textile with other materials.

| Type of conductive materials | Patterning techniques | Original electrical performance | Electrical performance after mechanical stress | Self-healable | Ref. |
| --- | --- | --- | --- | --- | --- |
| Cu serpentine mesh | Photolithography | 5.8 × 10^5^ S/cm | Not change significantly with 10% stretching | 🗴 | [1] |
| Cu mesh | Direct sputtering | 0.5-2 Ω/cm | / | 🗴 | [2] |
| Cu-Ag50 amalgam/textile | Embroidery | 2.4 × 10^6^ S/m | / | 🗴 | [3] |
| Silver ink | Screen printing | 16 (± 3) mΩ/sq | 17 (± 3) mΩ/sq  (1000 bending cycles) | 🗴 | [4] |
| Silver nanoflakes | Screen printing | 6.02 × 10^6^ S/m | 2 times of the original resistance  (1000 bending cycles) | 🗴 | [5] |
| AgNW | Solution casting and transfer | 8130 S/cm | / | 🗴 | [6] |
| LM-textile | “Dual mask” printing | 10 mΩ/sq | 8.4 mΩ/sq  (1000 bending cycles) | 🗸 | This work |

Table S2 The conductive material, manufacturing technique, and electromagnetic performance comparisons to other RF devices.

| Flex-ible | Conductive material | | Self-healable | Manufacturing  technique | Insertion loss  (mechnical test) | Gain (dBi) | Wireless communication on human body | Frequency (GHz) | Ref. |
| --- | --- | --- | --- | --- | --- | --- | --- | --- | --- |
| 🗸 | Conductive Non-woven Fabric 4770 | | 🗴 | Laser cutting | < 1.5 dB  (< 2 dB) | / | 🗸 | 2.45 | Spoof surface plasmonic waveguide ^[7]^ |
| 🗸 | Cu | | 🗴 | Photolithography | 0.9 dB cm^-1^  (🗴) | / | 🗴 | 28 | Microstrip transmission Line ^[8]^ |
| 🗸 | | Serpentine metal structure | 🗴 | Laser cutting | < 2 dB  (<2 dB) | / | 🗴 | 2-6 | Coplanar waveguide ^[9]^ |
| 🗴 | | SWCNT composite | 🗴 | Printing, cutting | 2.6 dB cm^-1^  (🗴) | / | 🗴 | 26 | Microstrip transmission Line ^[10]^ |
| 🗸 | | Cu | 🗴 | Photolithography | / | 7.41  🗴 | 🗴 | 26 | Antenna ^[8]^ |
| 🗸 | | Ti/Cu | 🗴 | Photolithography, magnetron sputtering | / | 9.6  🗴 | 🗴 | 28 | 1*4 array antenna ^[11]^ |
| 🗸 | | Copper strands | 🗴 | Embroidery | / | 4.23  🗴 | 🗴 | 1.5 | 1*2 array antenna ^[12]^ |
| 🗸 | | Cu/Ag50 amalgam | 🗴 | Embroidery | / | 6.5  🗴 | 🗴 | 2.47 | Antenna^[13]^ |
| 🗸 | | Silver | 🗴 | Screen printing | / | 7.33  🗴 | 🗴 | 2.4 | Antenna ^[14]^ |
| 🗸 | | LM | 🗸 | Microfluidic | / | / | 🗸 | 13.56*10^-3^ | Antenna ^[15]^ |
| 🗸 | | LM | 🗸 | Digital embroidery | / | / | 🗸 | 13.56*10^-3^ | Antenna ^[16]^ |
| 🗴 | | LM | 🗸 | Microfluidic | / | 5.07  🗴 | 🗴 | 2.5 | Antenna ^[17]^ |
| 🗸 | | Functionalized nanodroplets | 🗸 | a “dual-mask” printing | 0.0245 /0.351  dB mm^-1^  (0.03 /0.358  dBmm^-1^) | / | 🗸 | 2.45/24.5 | This work  (Microstrip transmission Line) |
| 🗸 | | Functionalized nanodroplets | 🗸 | a “dual-mask” printing | / | 10.67  (9.65) | 🗸 | 26 | This work  (2*2 array antenna) |

Table S3 The SAR values and realized gains of the LM-textile mmWave antenna array at 26 GHz.

| Human issue | 1 g SAR  (W/kg) | 10 g SAR  (W/kg) | Realized gain (dBi)  26 GHz |
| --- | --- | --- | --- |
| head | 0.472423 | 0.09745 | 6.963 |
| fat | 0.181343 | 0.11311 | 12.12 |
| Skin | 0.717027 | 0.346965 | 7.966 |
| muscle | 0.351805 | 0.16333 | 8.158 |

**References**

[1] T. Chang, Y. Tanabe, C. Wojcik, et al, “A General Strategy for Stretchable Microwave Antenna Systems using Serpentine Mesh Layouts,” Advanced Functional Materials 27, (2017): 1703059.

[2] H. Chen, S. Tang, W. Tian, et al, “Direct Sputtering on PDMS for investigation of

Stretchable and Transparent Microstrip Line,” Transactions on Components, Packaging and Manufacturing Technology 9, (2019): 1741-1747.

[3] D. Vital, S. Bhardwaj, J. Volakis, “Textile-Based Large Area RF-Power Harvesting System for Wearable Applications,” IEEE Transactions on Antennas and Propagation 68, no. 3 (2020): 2323-2331.

[4] J. Lee, P. Dzagbletey, M. Jang, J. Chung, J. So, “Flat Yarn Fabric Substrates for Screen-Printed Conductive Textiles,” Advanced Engineering Materials 34, no. 12 (2020): 2000722.

[5] H. Hong, J. Hu, X. Yan. “UV Curable Conductive Ink for the Fabrication of Textile-Based Conductive Circuits and Wearable UHF RFID Tags,” ACS Applied Materials & Interfaces 11, (2019): 27318-27326.

[6] L. Song, A. Myers, J. Adams, et al, “Stretchable and Reversibly Deformable Radio

Frequency Antennas Based on Silver Nanowires,” ACS Applied Materials & Interfaces

6 (2014): 4248-4253.

[7] Xi. Tian, Q. Zeng, S. Kurt, et al, “Implant-to-Implant Wireless Networking with Metamaterial Textiles,” *Nature Communications* 14, no. 1 (2023): 4335.

[8] M. Wagih, G. Hilton, A. Weddell, S. Beeby, “Broadband Millimeter-Wave Textile-based Flexible Rectenna for Wearable Energy Harvesting,” *IEEE Transactions on Microwave Theory and Techniques* 68, no. 11 (2020): 4960-4972.

[9] L. Ju, Z. Liu, B. Yu, H. Chen, Z. Xiao, W. Lu, “Stretchable and Dynamically Tunable Attenuator Based on Graphene,” *IEEE Transactions on Microwave Theory and Techniques* 70, no. 6 (2022): 2999-3008.

[10] A. Mehdipour, I. D. Rosca, A. Sebak, C. W. Trueman, S. V. Hoa, “Carbon Nanotube Composites for Wideband Millimeter-Wave Antenna Applications,” *IEEE Transactions on Antennas and Propagation* 59, no. 10 (2011): 3572-3578.

[11] F. Wang, J. Zhou, Y. Li, X. Xu, “Millimeter-Wave Patch Antennas with Ultralow Profile of 20 µM on Flexible Substrate,” *IEEE Transactions on Antennas and Propagation* 71, no. 3 (2023): 2339-2349.

[12] K. Zhang, L. Zheng, F. Farha, et al, “Three-Dimensional Textile Structural Conical Conformal Microstrip Antennas for Multifunctional Flexible Electronics,” *ACS Applied Electronic Materials* 2, (2020): 1440-1448.

[13] D. Vital, S. Bhardwaj, J. Volakis, “Textile-Based Large Area RF-Power Harvesting System for Wearable Applications,” *IEEE Transactions on Antennas and Propagation* 68, no. 3 (2020): 2323-2331.

[14] J. Lee, P. Dzagbletey, M. Jang, J. Chung, J. So, “Flat Yarn Fabric Substrates for Screen-Printed Conductive Textiles,” *Advanced Engineering Materials* 22, (2020): 2000722.

[15] K. Yamagishi, W. Zhou, T. Ching, S. Huang, M. Hashimoto, “Ultra-Deformable and Tissue-Adhesive Liquid Metal Antennas with High Wireless Powering Efficiency,” *Advanced Mater*ials 33, (2021): 2008062-15.

[16] R. Lin, H. Kim, S. Achavananthadith, et al, “Digitally-Embroidered Liquid Metal Electronic Textiles for Wearable Wireless Systems,” *Nature Communications* 13, (2022): 2190.

[17] L. Li, X. Yan, H. Zhang, Q. Wang, “Polarization-and Frequency-Reconfigurable Patch Antenna Using Gravity-Controlled Liquid Metal,” *IEEE Transactions on Circuits and Systems II: Express Briefs* 69, 3 (2022): 1029-1033.
